# Strategies to gain novel Alzheimer’s disease diagnostics and therapeutics using modulators of ABCA transporters

**DOI:** 10.17879/freeneuropathology-2021-3528

**Published:** 2021-12-13

**Authors:** Jens Pahnke, Pablo Bascuñana, Mirjam Brackhan, Katja Stefan, Vigneshwaran Namasivayam, Radosveta Koldamova, Jingyun Wu, Luisa Möhle, Sven Marcel Stefan

**Affiliations:** 1 Department of Pathology, Section of Neuropathology, Translational Neurodegeneration Research and Neuropathology Lab, University of Oslo and Oslo University Hospital, Oslo, Norway; 2 LIED, University of Lübeck, Lübeck, Germany; 3 Department of Pharmacology, Faculty of Medicine, University of Latvia, Rīga, Latvia; 4 Department of Pharmaceutical and Cellbiological Chemistry, Pharmaceutical Institute, University of Bonn, Bonn, Germany; 5 Department of Environmental and Occupational Health, School of Public Health, University of Pittsburgh, Pittsburgh, PA, United States of America

**Keywords:** ABC transporter, ABCB_1_ (P-gp), ABCC_1_ (MRP_1_), ABCG_2_ (BCRP), ABCA_1_ (ABC_1_), ABCA_2_, ABCA_5_, ABCA_7_, Multitarget inhibitor (PANABC), Broad-spectrum modulator, Alzheimer’s disease, Amyloid-beta (Aβ / Abeta), Inhibition, Activation, Induction, Downregulation, PET Tracer (PETABC), Pattern analysis, Polypharmacology, Rational drug design and development

## Abstract

Adenosine-triphosphate-(ATP)-binding cassette (ABC) transport proteins are ubiquitously present membrane-bound efflux pumps that distribute endo- and xenobiotics across intra- and intercellular barriers. Discovered over 40 years ago, ABC transporters have been identified as key players in various human diseases, such as multidrug-resistant cancer and atherosclerosis, but also neurodegenerative diseases, such as Alzheimer’s disease (AD). Most prominent and well-studied are ABCB1, ABCC1, and ABCG2, not only due to their contribution to the multidrug resistance (MDR) phenotype in cancer, but also due to their contribution to AD. However, our understanding of other ABC transporters is limited, and most of the 49 human ABC transporters have been largely neglected as potential targets for novel small-molecule drugs. This is especially true for the ABCA subfamily, which contains several members known to play a role in AD initiation and progression. This review provides up-to-date information on the proposed functional background and pathological role of ABCA transporters in AD. We also provide an overview of small-molecules shown to interact with ABCA transporters as well as potential *in silico*, *in vitro*, and *in vivo* methodologies to gain novel templates for the development of innovative ABC transporter-targeting diagnostics and therapeutics.

## INTRODUCTION

### From MDR to neurodegeneration: ABC transporters in human disease

ABC transporters are membrane-bound transport proteins that are ubiquitously present in the human body.^1-4^ They play a major role in determining the distribution of intrinsic and xenobiotic drugs between intra- and intercellular compartments.^5,6^ The clinical relevance of ABC transporters became pronounced when their expression was correlated to cross-resistance of cancer cells to antineoplastic agents.^3,7-13^ This phenomenon is called ‘multidrug resistance’ (MDR). However, despite enormous efforts and countless clinical trials to target these efflux pumps,^14-17^ MDR is still a major unresolved obstacle in cancer chemotherapy. To date, most ABC transporters have been associated with MDR,^3,7-9,11,12^ but only a small minority has been studied properly and can be addressed by small-molecule modulators.^18-22^ Amongst these are ABCB1,^1,18-27^ ABCC1,^1,18,19,23,24,26,27^ and ABCG2.^18,19,25^

Apart from their role in multidrug-resistant cancer, many ABC transporters have been identified as key players in neurological disorders. Evidence for this includes their high abundance at the blood-brain barrier (BBB) and blood-cerebrospinal fluid barrier (BCSFB) in the central nervous system (CNS).^28-32^ Additionally, their expression is altered in many pathological conditions in the brain.^28-30,33-40^ Important players are, again, ABCB1,^28-30,34-36,39-44^ ABCC1,^28-30,39,41,43,45^ and ABCG2^28,30,34,36,39-41,43^ in diseases like AD,^28-30,41^ amyotrophic lateral sclerosis (ALS),^34,36,44^ encephalopathy,^45,46^ epilepsy,^39,40^ multiple sclerosis (MS),^35^ and Parkinson’s disease (PD).^42,47^ Furthermore, ABC transporters were also found to be associated with certain genetic neurological and psychiatric diseases such as Huntington’s disease (HD),^38^ bipolar disorder,^48,49^ depression,^48^ or schizophrenia.^48,49^ Table 1 summarizes the involvement of ABC transporters in neurological diseases.

### ABC transporters, Aβ proteins, and AD

Since 2001, ABC transporters have been implicated in AD pathogenesis.^28-30,41,43,94,95^ Specifically ABCB1,^94^ ABCC1,^96^ and ABCG2^97^ have been suggested to directly transport amyloid-β (Aβ) proteins, being involved in Aβ clearance from the brain to the blood stream.^94,96,97^ In light of the failure of the first immunological treatment studies,^98^ it was already proposed that ABC transporter dysfunction could explain the clearance problem of Aβ.^99,100^ Cerebral accumulation of Aβ proteins interferes with neuronal metabolite homeostasis and leads to interruption of cortico-cortical circuits and hampered synaptic communication. This results in an irreversible atrophy and degeneration of specific brain regions, which further causes behavioral, cognitive, and visuospatial impairments in the progression of AD.^101^

The most prominent ABC transporter subfamily involved in AD is the ABCA subfamily of cholesterol and phospholipid transporters, in which particularly ABCA1, ABCA2, ABCA5, and ABCA7 have been associated with AD.^28-30,41,43,95,102^ For ABCA1,^28,41,95,103^ and specifically for ABCA7,^28,41,95,104-107^ genetic variant^28,41,108-111^ and genome-wide association studies (GWAS)^28,41,106,107,112^ have suggested that these transporters are risk factors in AD. These discoveries give the members of the ABCA subfamily a special standing within the group of AD-related ABC transporters.

Cholesterol metabolism in the context of AD has been discussed extensively before.^95,102,104,105,113-116^ The contribution of cholesterol and phosphilipid transport to membrane constitution, composition, fluidity, and lipid raft formation mediated by ABCA transporters has already been proposed,^6^ presenting a putative pharmacological target.^117^ Targeting cholesterol and lipid distribution impacts Aβ production by differential activities between α-, β-, and γ-secretases, but also amyloid precursor protein (APP) processing^106,118-122^ and Aβ degradation.^106,119,123-126^ A contribution of ABCA transporters to Aβ clearance from the brain was also proposed,^103,106,119,124,127^ but not through direct Aβ transport.^128,129^

Although ABCA transporters have been reviewed for the last two decades,^3,130,131^ little is known about their specific contribution to AD pathogenesis and their mode of action. This is mainly due to a lack of small-molecules that can be used to track, study, and impact the function of these under-studied ABC transporters.

The present review consists of two parts: **PART I** provides the *status quo* of ABCA transporters in AD and small-molecule modulators – in particular intrinsic substrates, natural compounds, pharmacological drugs, and synthetic molecules – that have been reported to influence ABCA transporter function and expression; **PART II** outlines the necessary drug development pipeline for the discovery of novel lead structures as potential innovative diagnostics and therapeutics against AD. This pipeline includes cutting-edge *in silico* methodologies, established *in vitro* cell assays, and necessary *in vivo* models.

Collectively, this review contributes to a deeper understanding of small-molecule ligands that influence ABCA transporter function, potentially leading to the development of novel AD diagnostics and therapeutics.

## PART I: *STATUS QUO*

### ABCA transporters: Physiological function and implications for AD

ABCA transporters are ubiquitously present in the human body,^3,10,13^ although differentially expressed.^10^ All of the 12 subfamily members have been associated with cholesterol and/or phospholipid transport and homeostasis,^3,13,132^ except for ABCA4, which is primarily a transporter of retinoids.^133-138^

In addition to the diseases listed in Table 1, ABCA transporters have been described as key proteins in several other human disorders, including neonatal respiratory distress syndrome (ABCA3),^139^ chronic interstitial lung disease (ABCA3),^140^ cataract-microcornea syndrome (ABCA3),^141^ hypertrichosis terminalis (ABCA5),^142^ or Harlequin ichtyosis (ABCA12).^143^

However, one major clinical implication for ABCA transporters, particularly ABCA1, ABCA2, ABCA5, and ABCA7, relates to AD.^28,50,52,63^ Their suggested roles in this major burdensome neurodegenerative disease as well as general physiological aspects are summarized in the following sections.

#### ABCA1

ABCA1 is the prototype of the ABCA subfamily,^144^ was first identified in 1994, and is located on human chromosome 9.^145^ The complete genomic sequence of human *ABCA1* was reported in 2000. The *ABCA1* gene spans 149 kb comprising 50 exons, and the resulting protein is 2261 amino acids long.^146^ ABCA1 is located in the plasma membrane and is also present intracellularly in the endoplasmic reticulum and Golgi apparatus, where it mediates the efflux of cholesterol and phospholipids from intracellular compartments to extracellular lipid-free apolipoproteins, mainly apolipoprotein A1 (APOA1) and to a lesser extend APOA2 and APOE, to form high-density lipoprotein (HDL) particles.^3,147,148^ The lipidation of APOA1 is preceded by ABCA1 dimerization.^149^ ABCA1 thus represents the first and rate-limiting step in the reverse cholesterol transport pathway, which removes excess cholesterol from peripheral tissues *via* HDL and delivers it to the liver for conversion into bile acids and subsequent excretion. In contrast to peripheral tissues, the physiological role of ABCA1 in the brain, where it is expressed in all cell types, is not well defined.^103^ It has been suggested that ABCA1 is required for cholesterol transport from glial cells to neurons *via* APOE, which is secreted by glial cells and serves as the main lipid acceptor in the brain.^103,125^ *In vitro* and *in vivo* studies in *Abca1* knock-out models demonstrated that ABCA1 is essential for normal APOE secretion and lipidation in the CNS.^150,151^ Glial cells deficient for ABCA1 showed reduced lipid efflux with concurrent lipid accumulation as well as decreased APOE secretion, with APOE particles being small and poorly lipidated. In mice, *Abca1* knock-out resulted in dramatically decreased brain levels of APOE. Moreover, examination of the hippocampi of *Abca1*-deficient mice revealed a decrease in neurite length and number of neurite segments and branches, pointing to an importance of ABCA1 for neurite integrity.^152^

The major genetic risk factor for sporadic AD is the allelic state of the *APOE* genotype, with inheritance of the *APOE4* allele markedly increasing disease risk.^153,154^ Recently, Rawat *et al.* investigated how *APOE4* affected ABCA1 expression and function *in vitro* in astrocytes.^155^ The authors found that *APOE4* decreased ABCA1 plasma membrane levels and increased ABCA1 co-localization with late endosomes *via* activation of ADP-ribosylation factor 6, thereby reducing cholesterol efflux and lipidation of APOE particles. They corroborated their findings in blood-cerebrospinal fluid (CSF) showing that CSF from homozygous carriers of the *APOE4* allele was less efficient in stimulating ABCA1-mediated cholesterol efflux compared to CSF from homozygous carriers of the *APOE3* allele.

A recent study assessed cholesterol efflux capacity of CSF by analyzing AD patients, non-AD patients, and control subjects.^156^ The results demonstrated that ABCA1-mediated CSF-cholesterol efflux capacity was markedly reduced in AD but not in non-AD demented patients. However, this difference did not depend on APOE4 status. Interestingly, ABCA1-mediated CSF-cholesterol efflux capacity inversely correlated with total and phosphorylated protein tau, suggesting a link between the dysfunction of HDL-like particle in CSF and neurodegeneration.

Apart from the indirect link *via* APOE, a direct link between ABCA1 and AD has also been subject to investigation. Expression of hippocampal ABCA1 was elevated on both the mRNA and protein levels and was positively correlated with neuropathological changes and dementia severity in AD patients.^157^ The authors of this study suggested that the observed upregulation of ABCA1 could be interpreted as a compensatory attempt to clear Aβ from the brain. Moreover, a variety of studies investigated associations between single nucleotide polymorphisms (SNP) in the *ABCA1* gene and the risk for ad,^28,108-111^ reporting inconclusive results.^95,103^ A meta-analysis of several studies identified the *ABCA1* rs2422493 (C477T) polymorphism as a risk factor for AD while no association was found for the rs2066718 (V771M) or rs1800977 (C14T) polymorphisms.^111^ This risk effect for rs2422493 was confirmed in a recent genetic variant association study that, in contrast to the meta-analysis, also reported an increased AD risk for rs2066718 and a decreased AD risk for rs1800977.^109^ Further genetic association studies and meta-analyses are necessary to search for potential associations between *ABCA1* polymorphisms and AD risk.

In a recent AD GWAS, the rs1800978 polymorphism in the *ABCA1* gene was identified as the lead SNP in a new genome-wide significant locus.^158^ The association of genetic variants of the *ABCA1* gene with AD risk was confirmed by exome sequencing data analysis from 32,558 individuals.^158^ The study identified around 120 variants that have an increased frequency in early-onset AD (EOAD; 1.5%) and late-onset AD (LOAD; 1.1%) cases, compared to 0.5% of all controls. The data demonstrated that AD-association was mainly explained by extremely rare variants, but also by a smaller number of more common variants, *e.g.*, N1800H.^159^ Intriguingly, loss of function and missense variants in the *ABCA1* gene were respectively associated with a 4.7-fold (95%CI 2.2-10.3) and 2.7-fold (95%CI 1.9-3.8) increased EOAD risk, and this was lower for LOAD cases suggesting that the burden of damaging ABCA1 variants was concentrated in younger AD patients.

Additionally, some long non-coding (lnc) RNAs such as lncRNA *LOC286367* have been shown to affect ABCA1 expression.^160^ LncRNA *LOC286367* and *ABCA1* are located on the same chromosome but are transcribed in opposite directions. A recent study demonstrated that *LOC286367* reduces ABCA1 expression in THP-1 macrophages and increases the levels of proinflammatory cytokines.^160^

The role of ABCA1 in Aβ deposition and clearance as well as in Aβ deposits-related memory deficits has been extensively investigated in *APP*-transgenic mouse models of AD. The lack of ABCA1 decreased brain APOE levels and either did not affect or increased Aβ load.^161-163^ A recent study utilizing shotgun lipidomics experiments demonstrated a common APOE isoform-specific phospholipid signature between human *APOE3/3* and *APOE4/4* AD brains and lipoproteins isolated from astrocyte-conditioned media of *APOE3* and *APOE4* mice.^164^ Interestingly, the lipoproteins derived from wild-type and *Abca1*^het^ mice had phospholipid content *APOE3* > *APOE4* > *APOE3^het^* > *APOE4^het^* suggesting that the combination of ABCA1 insufficiency and *APOE4* genotype decreases APOE lipidation even further, thus aggravating APOE4 effect. These findings suggest that poorly lipidated APOE may promote Aβ aggregation.^129,161-163^ In contrast, overexpression of ABCA1 in an *APP*-transgenic mouse model resulted in increased lipidation, albeit reduced brain levels of APOE and decreased Aβ load, implying that highly lipidated APOE may reduce Aβ aggregation propensity.^127^ This is supported by findings of Deane *et al.*, who showed that different APOE isoforms may differentially disrupt Aβ clearance from mice brains.^165^ A stable isotope-labelling kinetic study in an *APP*-transgenic mouse model either lacking *ABCA1* or overexpressing ABCA1 demonstrated increased APOE clearance in both *Abca1* knock-out and ABCA1-overexpressing mice, but did not reveal any effect on Aβ clearance or production, suggesting that ABCA1 may regulate Aβ deposition by a mechanism other than altering Aβ metabolism.^166^ In contrast, a study assessing the clearance of intracerebrally injected ^125^I-Aβ from the brain reported that *Abca1*-deficiency decreased Aβ clearance in non-*APP*-transgenic mice.^167^ Furthermore, knock-out of *Abca1* was found to augment the dissemination of intracerebrally injected, brain-derived Aβ seeds in *APP*-transgenic mice.^167^ Haplodeficiency of *Abca1* led to decreased brain APOE levels and increased Aβ oligomer levels but did not affect Aβ deposition in *APP*-transgenic mice.^168^ However, both haplodeficiency and homozygous knock-out of *Abca1* aggravated cognitive deficits in *APP*-transgenic mice.^152,167,168^ Lastly, the lack of one copy of *Abca1* exacerbated memory deficits, decreased Aβ clearance, and increased Aβ load in APP-transgenic mice expressing human APOE4 but not in *APP*-transgenic mice expressing human APOE3.^169^

#### ABCA2

ABCA2 is predominantly, but not exclusively, expressed in the brain, where it can be found in glial cells and neurons.^170-173^ On the subcellular level, ABCA2 is located in endo- and lysosomal membranes, facilitating the sequestration of waste substances into intracellular vesicles.^172^ In addition, it is involved in myelin lipid transport, neural development, and macrophage activation.^30,174,175^

Genetic variations of *ABCA2* were identified as a risk factor for EOAD and sporadic AD.^52,176^ These two studies showed a strong correlation between rs908832 and AD.^52,176^ However, a later study could not find a link between this SNP and any form of AD.^177^ In addition, *ABCA2* mRNA expression was upregulated in AD patients compared to controls suggesting ABCA2 as a biomarker for differential diagnosis of AD.^178^ Preclinical studies of ABCA2 suggested that this transporter modulates Aβ production *via* the LDL receptor (LDLR).^179,180^ ABCA2 overexpression increased LDLR density, and LDLR deficiency has been described to enhance Aβ deposition.^181^ Chen *et al.* reported a co-localization of ABCA2 and Aβ as well as Aβ upregulation in cells overexpressing ABCA2. In addition, impairment of ABCA2 expression using small interfering RNA (siRNA) was accompanied by a decrease in Aβ production.^182^ *Abca2* depletion has been shown to induce a shift from β- to α-secretases and thus, a reduction of APP processing by γ-secretase.^182^ Furthermore, ABCA2 has been proposed to play a role in Aβ production as it has been reported to upregulate sphingosine in murine cells and, therefore, to induce *APP* transcription.^183^ However, another study in human cells could not confirm the modulation of Aβ production or cholesterol efflux by ABCA2.^184^ Thus, further research on the role of ABCA2 in AD pathogenesis and its potential as a therapeutic target is necessary.

#### ABCA3

Despite its initial report of exclusive lung expression,^185^ ABCA3 is also found in other tissues including the brain.^186,187^ Within the brain, the highest levels of ABCA3 were found in oligodendrocytes.^188^

ABCA3 plays a role in producing surfactants in the lung, suggesting that the transporter may also be involved in lipid metabolism in the brain, specifically phosphatidylcholine and phosphatidylglycerol transport. Interestingly, phosphatidylcholine has also been discussed in the context of AD.^189^ A genetic study revealed that mutations in *ABCA3* can also cause cataract-microcornea syndrome, a rare congenital malformation of the eye.^141^ The actual implications of the potential connection between altered ABCA3 functionality and AD need to be addressed in future studies.

#### ABCA4

ABCA4 is mainly expressed in the retina with very little presence in other tissues of the CNS.^190^ *ABCA4* mutation causes Stargardt disease, characterized by macular dystrophy, retinal alterations, and lipofuscin accumulation.^60,61,190,191^ Other retinal diseases, such as fundus flavimaculatus, retinitis pigmentosa, or cone-rod dystrophy, have also been associated with mutations of *ABCA4*.^55,57,58,192^ ABCA4 is expressed in brain capillary endothelial cells, as well.^193^ However, no link between ABCA4 and AD has been suggested to date.

#### ABCA5

ABCA5 is a little-known member of the ABCA subfamily expressed mainly in skeletal muscle with unknown function in the brain.^194^ Studies in peripheral tissues suggest that the function of ABCA5 is associated with cellular lipid metabolism.^195^ *Abca5* knock-out in mice induced signs of lysosomal storage disease in the heart and the thyroid gland.^131^

In the brain, ABCA5 is expressed in neurons and, to a lesser extent, in microglia, astrocytes, and oligodendrocytes.^195^ Fu *et al.* showed that ABCA5 stimulated cholesterol efflux in neurons and induced a decrease in Aβ production probably affecting APP processing but not its expression.^195^

#### ABCA6

ABCA6 is ubiquitously expressed with high levels in liver, lung, heart, brain, and ovaries. This transporter is probably involved in macrophage lipid homeostasis as it is upregulated during macrophage differentiation and is responsive to cholesterol treatment.^196^ Although certain missense variants of *ABCA6* have been correlated with blood cholesterol levels,^197^ no link between ABCA6 and AD has yet been found.

#### ABCA7

*ABCA7* was first identified in the year 2000, and is located on human chromosome 19.^198-200^ Analysis of *ABCA7* mRNA expression levels has shown that this transporter is mainly confined to the brain and the immune system.^3^ Due to its high homology to *ABCA1* (54%),^200^ ABCA7 was first hypothesized to play an important role in lipid trafficking, mediating cholesterol and phospholipid efflux. ABCA7 actively transports phosphatidylcholine, phosphatidylserine, and sphingomyelin from the cytoplasm to the exocytoplasmic leaflet of membranes.^198,199,201^ However, in contrast to ABCA1, ABCA7 generates only small HDL particles.^202^ Recent research has shown that lipid trafficking by ABCA7 plays a secondary role. Studies in *Abca7* knock-out models have demonstrated that ABCA7 is involved in the phagocytotic activity of macrophages and fibroblasts^198,203-205^ but not in cell cholesterol release.^206-208^

In 2011, Hollingworth *et al.* identified the *ABCA7* gene as an AD risk locus.^198,209^ In multiple studies, variants of *ABCA7* have been associated with an increased risk of developing AD.^198,210-212^ In 2015, Steinberg *et al.* reported that rare loss-of-function variants of *ABCA7* confer a risk of AD in Icelanders (odds ratio: 2.12; *P* = 2.2 · 10^−13^), and found a similar association in study groups from Europe and the United States (combined odds ratio: 2.03; *P* = 6.8 · 10^−15^).^213^ In particular, the rare AD-related polymorphism rs200538373 was associated with an AD risk odds ratio of 1.9.^210^ These studies suggest that reduced levels of ABCA7 may increase the risk of AD. Nonetheless, it is not clear how these polymorphisms affect ABCA7 function and contribute to AD progression. Increased levels of ABCA7 expression were described in AD patients and were also positively correlated with cognitive decline.^198,211^ This finding is consistent with *Abca7* mRNA transcription levels in J20 mice.^123^ The increase of ABCA7 may be a compensatory defense mechanism that is insufficient to stop disease progression. Furthermore, the rs3764650G allele has been associated with increased neuritic plaques in human patients^198,214^ and a limitation of the neuroprotective effects of exercise intervention.^215^ These studies support a potential protective role of ABCA7 in AD. To date, three potential roles have been identified for ABCA7 contribution to AD: APP processing, immune response, and lipid metabolism.

Chan *et al.* proposed an inhibitory effect of ABCA7 on Aβ deposition after showing *in vitro* inhibition of Aβ production independent of β-secretase activity.^120^ Other authors proposed that ABCA7 is not directly linked to Aβ production, but rather through lipid metabolism as ABCA7 mediates the transport of lipids across the BBB and ABCA7 loss of function may alter cholesterol transport by decreasing APOE secretion and ABCA1 expression. This alteration in cholesterol metabolism can also contribute to AD development.^216^ However, *Abca7* knock-out induced an increase of Aβ load with no difference in clearance rate and an increase of β-secretase expression. On the other hand, ABCA7 overexpression led to diminished Aβ production and improved cognitive function.^217,218^

Nevertheless, ABCA7 is highly expressed in phagocytic cells, including macrophages and microglia, suggesting a role of the transporter in phagocytosis.^188,198^ Phagocytosis is crucial to maintain brain homeostasis. Indeed, ineffective phagocytosis may induce neuroinflammation, which is a risk factor in AD. In addition, microglial cells are involved in phagocytosis and degradation of Aβ. Thus, an involvement of ABCA7 in microglial phagocytosis of Aβ may explain the contribution of this transporter to AD pathogenesis. In AD patients, increased *ABCA7* transcription has been found in areas with plaques but not in unaltered regions such as the cerebellum.^123^ This increase in transcription was paralleled by microglia recruitment supporting the contribution of ABCA7 to microglia-mediated phagocytosis of Aβ. In addition, *Abca7* knock-out mice showed a reduced microglia response after intracerebral Aβ injection.^123^ Kim *et al.* demonstrated an increased Aβ load in J20/A7 knock-out mice compared to J20 mice, potentially due to an altered phagocytic function.^124,198^ Furthermore, it has recently been shown that *Abca7* haplodeficiency disturbs the microglial immune response and causes enhanced Aβ accumulation in microglia, probably due to alterations in endolysosomal trafficking.^219^

Last, a new hypothesis has emerged recently, assigning ABCA7 a prominent role in the altered lipidostasis hypothesis in AD.^104^ The authors of this study proposed the existence of a neurodegenerative lipid that is naturally removed by ABCA7. A loss of ABCA7 function due to the described polymorphisms might accelerate accumulation of this lipid, inducing Aβ aggregation. In fact, a link between cholesterol metabolism and ABCA7-mediated phagocytosis has been reported, which may also explain the protective properties of statin treatment in the development of AD.^105,198,203,220^

Despite recent findings, the role of ABCA7 in AD pathogenesis remains unclear. According to *in vitro* and preclinical research, it may be associated with phagocytic activity by microglia, which could be linked to cell cholesterol metabolism.^105,198,203^ Thus, further investigation is required to reveal the role of ABCA7 in AD pathogenesis and its potential use as a therapeutic target for this neurodegenerative disease.

#### ABCA8–ABCA10

So far, no obvious role of ABCA8–10 has been elucidated for AD, neurodegenerative diseases, nor any human disease. However, several potential intrinsic substrates of ABCA8 have been identified.^10,221,222^ Furthermore, a significant number of ABCA transporter modulators have been identified on this target.^222^ Hence, ABCA8 represents a good model system for the development of potential therapeutics targeting other ABCA transporters taking the scarce knowledge on this transporter subclass into account.

#### ABCA12

ABCA12 is expressed predominantly in the epidermis, and its main function is the transport of lipids.^223^ It is hypothesized that ABCA12 plays a role in skin lipid homeostasis. Mutations in this gene are associated with lamellar ichthyosis type 2 and Harlequin ichthyosis.^143,224,225^ However, a Japanese study investigated common polymorphisms of *ABCA12* and did not find an association with sporadic AD.^226^

#### ABCA13

ABCA13 is the largest ABC transporter with 576 kDa.^227^ It has been reported to be highly expressed in the brain as well as in peripheral tissues.^227^ A very small study found reduced neuroinflammation and altered ABCA13 expression in *post mortem* analyses of brains from patients with Lewy body dementia.^64^ In addition, increased ABCA13 expression has been reported after stroke in mice.^67^ Furthermore, two studies showed enhanced *ABCA13* mRNA expression in schizophrenic patients after different antipsychotic treatments, suggesting a role of this transporter in psychiatric disorders.^48,65,66^ However, no association between ABCA13 and AD has been found.

### Modulators of ABCA transporter function, trafficking, and regulation

‘Modulation’ is a widely used term to summarize actions of small-molecules that have been reported to alter ABCA transporter function, trafficking, and/or regulation. Modulators can be divided into ‘interactors’ and ‘regulators’.

**Figure 1.**
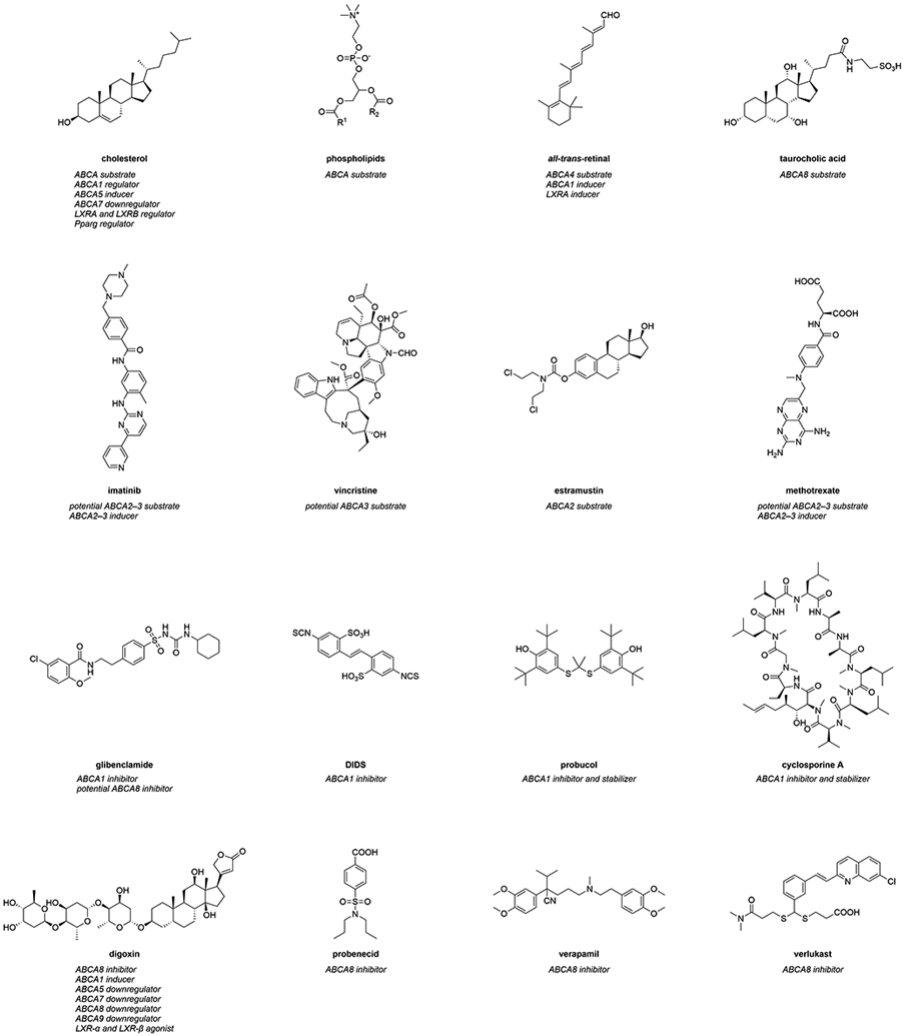
Molecular formulas of prominent interactors of ABCA transporters.

Interactors summarize compounds that directly bind to ABCA transports, which can have either inhibiting or activating effects on the transporters. Substrates are also included in this category. In terms of ABCA transporters, however, a direct interaction of these agents with their target(s) has in most cases not yet been comprehensively proven. Therefore, compounds that are believed to directly interact with ABCA transporters extend the category of interactors. Figure 1 represents the most prominent interactors of ABCA transporters and provides additional information about their mode of modulation.

**Figure 2.**
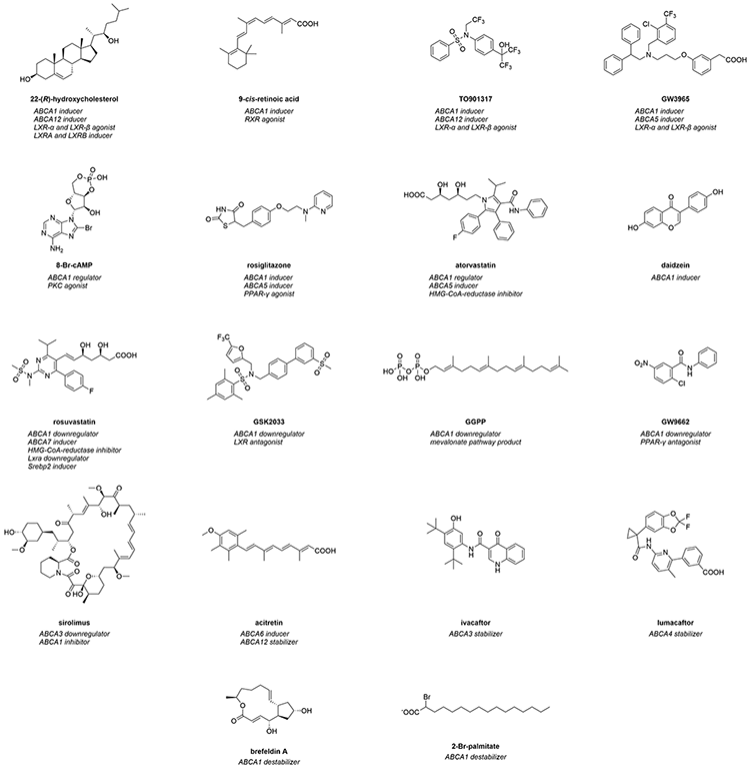
Molecular formulas of prominent regulators of ABCA transporters.

Regulators are compounds that change ABCA transporter expression (transcription and/or translation) in terms of induction and/or downregulation. In addition, compounds that regulate ABCA transporter trafficking can be included into the category of regulators, as this effect was often observed as ‘pseudo-protein increase’ at the cell membrane. Figure 2 depicts the most prominent regulators of ABCA transporters including proposed mode of modulations.

**Figure 3.**
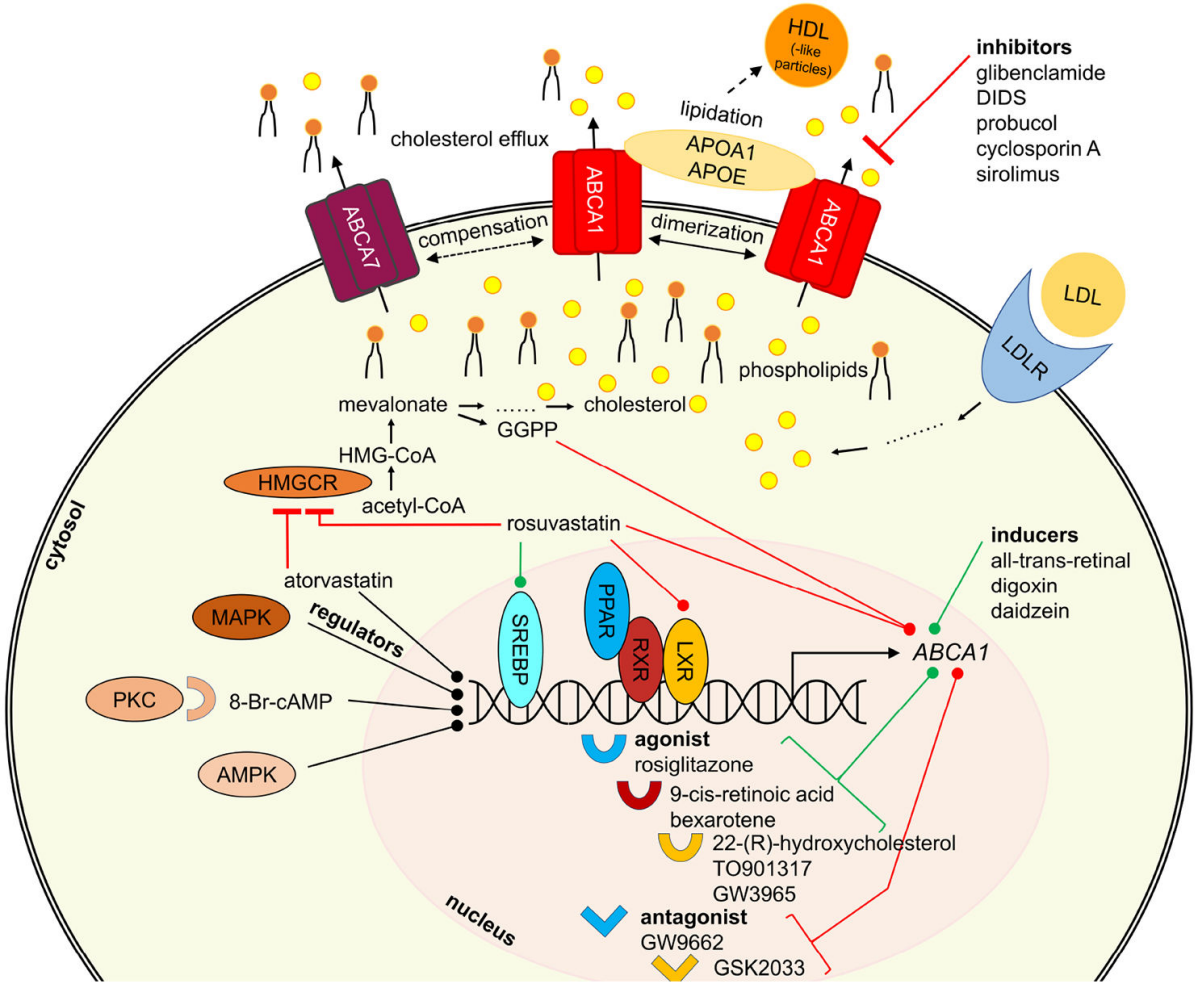
General overview of proteins participating in ABCA1 regulation and interaction.

It must be stated that the term ‘inhibitor’ and ‘activator’ are often misused in the literature, as in most cases studies describe a downregulation or induction. In the present review, this mislabeling has been taken into account and the present review and the respective compounds have been allocated into the correct groups. As established earlier,^23,24^ the compounds are sorted according to their origin: (i) intrinsic substrates and substrate-like molecules, (ii) (other) natural compounds, (iii) pharmacological drugs, (iv) high-throughput screening-(HTS)-derived candidates, as well as (v) compounds from synthetic/medicinal chemistry approaches. Figure 3 gives a general overview of specific interactors and their postulated mode of modulation. Table 2 summarizes all modulators of ABCA1, the most studied ABCA transporter, while Table 3 summarizes all known modulators in terms of the other ABCA transporters. The stated concentration values are indicators of bioactivities of the respective compound and are strongly dependent on the testing system utilized. Hence, the respective data must be interpreted with caution.

#### Small-molecule interactors of ABCA transporters

##### Endo- and xenobiotic substrates

The most genuine interactors of ABCA transporters are intrinsic substrates of these transporters. These include cholesterol (Figure 1) and other sterol derivatives,^10,221,222,228^ but also phospholipids (Figure 1), sphingolipids^228,229^ and retinoids (*e.g., all-trans*-retinal; Figure 1).^133-138^ In addition, certain intrinsic molecules were demonstrated to interact with ABCA transporters, in particular with ABCA1^230^ and ABCA8.^10,221,222^ α-tocopherol (vitamin E) was demonstrated to be transported by ABCA,^230^ and to interfere with *ABCA1* regulation.^231^ The sterol derivatives estradiol-β-glucuronide, estrone sulfate, and taurocholic acid (Figure 1), but also the physiological substrate leukotriene C4 (LTC_4_), the natural compound ochratoxin A, as well as the chemical *p*-amino hippuric acid were discovered as (potential) ABCA8 substrates.^10,221,222^ Specifically the ABCA8-mediated taurocholate export from various human pancreatic cancer cell lines was suggested as the major mechanism behind gemcitabine resistance in these cells,^221^ which was corroborated in HEK293 cells stably expressing ABCA8.^10^

In addition, a small body of evidence suggests that ABCA2 and ABCA3 contribute to the subcellular sequestration of certain antineoplastic agents into endo- and lysosomes.^232-235^ These agents include cytarabine (ABCA3),^235^ daunorubicin (ABCA3),^232,233,235^ etoposide (ABCA3),^235^ imatinib (ABCA2 and ABCA3; Figure 1),^234,236^ mitoxantrone (ABCA3),^235^ and vincristine (ABCA3; Figure 1).^235^ Furthermore, several antineoplastic agents were described to have less effect when ABCA2 was overexpressed *in vitro*^171,237,238^ and *in vivo*.^239^ For example, the anticancer drug estramustine (Figure 1) was effluxed from ABCA2-overexpressing human ovary carcinoma cells, which were less susceptible to estramustine treatment than the sensitive cell line.^171,238^ Antisense nucleotide treatment against ABCA2 re-sensitized the carcinoma cells, further demonstrating a role for ABCA2 in mediating drug efflux.^238^ Furthermore, *Abca2* knock-out mice had elevated estradiol and estrone levels when treated with estramustine.^239^ A similar effect in terms of susceptibility and resensitization was observed for ABCA3-mediated transport of miltefosine in *Leishmania*,^240^ doxorubicin resistance in acute myeloid leukemia cells,^237^ and cisplatin as well as paclitaxel resistance in several lung cancer cell lines.^241^

Strikingly, ABCA2 co-localized with the lysosomal-associated membrane protein 1 (LAMP1) – an endolysosomal marker – as well as the fluorescence probe dansyl-estramustine. This co-localization indicates a direct sequestration of this antineoplastic drug into endo- and/or lysosomes.^171^ On the other hand, the susceptibility of ABCA3-overexpressing CCRF-CEM leukemia cells to the antineoplastic agents cytarabine, methotrexate (Figure 1), vincristine, but also the anti-inflammatory drug dexamethasone, was reduced compared to their parental counterparts.^242^ Taken together, ABCA2 and ABCA3 are contributors to MDR, and the number of potential ABCA2 and ABCA3 substrates may be even higher than currently suggested.

Interestingly, missense mutations of *ABCA4* were associated with chloroquine- and hydroxychloroquine-associated retinopathy,^243^ although contradictory studies exist.^244^ A direct interaction was postulated, however, not proven. Nevertheless, these results suggest chloroquine and hydroxychloroquine as potential ABCA4 substrates.

##### Inhibitors

To date, the number of small-molecules that (are believed to) directly interact with ABCA transporters is very low. For example, only 14 inhibitors can be found in the literature regarding the most studied prototype of ABCA transporters, ABCA1.^245-248^ Only four of these inhibitors are associated with half-maximal inhibition concentrations (IC_50_),^245,249^ which is the ‘golden surrogate’ to evaluate and judge inhibitory activities of small-molecules. The following section will highlight these small-molecules as well as inhibitors of other ABCA transporters.

#### ABCA1

##### Glibenclamide and 4,4’-diisothiocyano-2,2’-stilbene-disulfonic acid (DIDS)

As outlined above, ABCA1 is the most studied and understood ABCA transporter, although its particular role in neurodegenerative diseases in general^51,103^ – and in AD in particular – is not well understood.^28-30,43,95,102^ However, over time, several agents were found to impact ABCA1 transport function. The most prominent examples are glibenclamide and DIDS (both Figure 1), which were first shown to inhibit ABCA1 in 1997.^247,248^ These drugs blocked the ABCA1-mediated ^125^I efflux from murine peritoneal macrophages^247^ as well as human ABCA1-transfected *Xenopus laevis Oocytes*.^248^ Glibenclamide and DIDS inhibited the ABCA1-mediated transport of cholesterol and other sterols as well as phospho- and sphingolipids. Thus, these agents became the ‘standard ABCA1 inhibitors’ and have frequently been used in ABCA1 studies ever since.^229,250-269^ Glibenclamide and DIDS were preferred over other discovered ABCA inhibitors, such as bumetanide, diphenylamine 2-carboxylic acid, flufenamic acid, furosemide, and bromosulfophthaleine.^248^ Specifically glibenclamide was rigorously evaluated regarding its mechanism of action. It was demonstrated that glibenclamide prevented cross-linking of ^125^-marked APOA1 to ABCA1,^267,270^ not interfering with ABCA1 location at the cell surface.^267^ In essence, glibenclamide and DIDS may play a significant role in the development of future modulators of ABCA transporters in general.

##### Probucol and cyclosporine A

Less prominent but also well characterized are the antilipidemic drug probucol^246,271-278^ and the immunosuppressant cyclosporine A^245,249,258,279-281^ (both Figure 1). Probucol was demonstrated to reduce the cholesterol efflux from different ABCA1-overexpressing murine and human macrophages,^275-278^ and total lipid release (cholesterol + phospholipids) from human WI-38 fibroblasts.^246^ Vice versa, probucol increased accumulation of free cholesterol, cholesterol esters, phosphatidylcholine, and sphingomyelin in human fibroblasts.^246^ Additionally, probucol was reported to prevent cell surface-specific binding of ^125^I-marked APOA1 to ABCA1.^246,278^ Similarly, this effect has already been demonstrated for glibenclamide before.^267,270^ Interestingly, it was shown that total ABCA1 protein levels were increased after exposure to probucol due to decreased degradation.^246,275^ This qualifies probucol also as a stabilizer. However, as its inhibiting effect is far more pronounced, we have included it as an inhibitor here.

The immunosuppressant cyclosporine A has been characterized as an ABCA1 inhibitor in multiple studies.^245,249,258,279-281^ This inhibition was shown to be direct through a radiolabeled variant of cyclosporine A and purified ABCA1.^245^ Cyclosporine A not only functionally inhibited ABCA1-mediated cholesterol and phospholipid efflux,^245,249^ and caused intracellular accumulation of cholesterol,^258^ but also inhibited the ABCA1-dependent binding of Alexa 546- or ^125^I-labeled APOA1,^245,249^ as demonstrated for glibenclamide^267,270^ and probucol^246,278^ before. Interestingly, toxicity assays demonstrated that cyclosporine A negated the positive effect of an *ABCA1* inducer on cell viability when cells were exposed to Aβ proteins.^280^ This was confirmed *in vivo* in C57BL/6 mice that had reduced HDL levels.^249^ Interestingly, cyclosporine A was shown to decrease ABCA1 turnover, increasing its presence at the cell surface by a factor of two as demonstrated with a GFP-labeled ABCA1 variant,^249^ suggesting a similar mode of inhibition as for probucol.^275^ Thus, as for probucol,^246,275^ cyclosporine A also appears to have a stabilizer function,^275^ but is included in the current section due to its pronounced inhibitory role. Morevover, the cyclosporine A analog valspodar (PSC833) inhibited direct binding of radiolabeled cyclosporine A to ABCA1, revealing that valspodar also acts as an ABCA1 inhibitor.^245,282^ Furthermore, several other calmodulin antagonists inhibited ABCA1-mediated cholesterol efflux and binding of APOA1.^245^ These include pimecrolimus,^245^ sirolimus,^245^ and tacrolimus,^245^ suggesting these molecules as potential scaffolds for the development of future ABCA1 modulators.

##### Other ABCA1 inhibitors

In terms of other small-molecules that were suggested to inhibit ABCA1 function, BLT-4 has been demonstrated to inhibit cholesterol and phospholipid export from adipocytes and macrophages,^255^ and to decrease cholesterol efflux from *ABCA1*-transfected HEK293 cells. BLT-4 was also shown to inhibit ^125^I-marked APOA1-binding to ABCA1,^270^ as demonstrated for glibenclamide,^267,270^ probucol,^246,278^ and cyclosporine.^245,249^

#### Other ABCA transporters

While ABCA1 can be considered a less-studied ABC transporter with certain knowledge about its function and interfering small-molecules,^18^ all other ABCA transporters belong to the group of under-studied ABC transporters that cannot be addressed by small-molecules with very rare exceptions.^18^

One rare example is ABCA8. Using the *Xenopus laevis Oocytes* model *in vitro* testing system,^248^ Tsuruoka *et al.* reported inhibitors of this transport protein.^222^ While digoxin, probenecid, and verapamil (all Figure 1) could be identified as very weak inhibitors of ABCA8-mediated estradiol-β-glucuronide transport, dofequidar (MS-209), ochratoxin A, and verlukast (MK-571; Figure 1) were discovered as moderately potent inhibitors.^222^ In addition, glibenclamide was also suggested to (partially) inhibit ABCA8 function.^266^

##### Activators

Although activators of ABC transporters have been reported, as for example, for ABCB1^23^ and ABCC transporters,^23,283-288^ these reports are somewhat scarce compared with other classified modulators of ABC transporters. In terms of A subclass ABC transporters, no small-molecule activators are known. However, it is well established and has been extensively demonstrated that ABCA1 activity depends on (co)-administration of HDL and/or APOA1.^117^ HDL and APOA1 are not small-molecules but peptides, and therefore fall outside of the scope of the present review. Similarly, it has been shown in several reports that HDL-mimics consisting of 26 amino acids are able to increase ABCA1-mediated transport.^289^ Although these molecules are also not small-molecules, the scarceness of activators of ABCA transporters warrants the inclusion of these middle-sized molecules here.

In 2004, structural elements of APOA1 were discovered to promote ABCA1-mediated cholesterol efflux.^290^ In 2007, Vedhachalam *et al.* discovered that the C-terminus of APOE promoted ABCA1-mediated efflux from murine J774.A1 macrophages.^291^ The latter discovery led to the development of two short-length peptides, ATI-5261 and CS-6253, consisting of 26 amino acids each.^289^ Their amino acid sequences expressed in single-letter code are EVRSKLEEWFAAFREFAEEFLARLKS^289^ and EVCitSKLEEWLAALCitELAEELLACitLKS (Cit = citrulline),^292^ respectively, which is of particular interest for the development of novel lead structures. Both peptides increased ABCA1-mediated cholesterol and phospholipid transport in murine and human macrophages.^289,292^ Interestingly, CS-6253 decreased ^125^I-labed APOA1 binding to ABCA1,^292^ as demonstrated for glibenclamide,^267,270^ probucol,^246,278^ cyclosporine A,^245,249^ and BLT-4^270^ before. However, CS-6253 was shown to compete with APOA1 to promote ABCA1-mediated transport.^292^ Both ATI-5261 and CS-6253 have a high practical relevance regarding AD and other neurodegenerative diseases, as these agents demonstrated *in vivo* efficacy.^289,293^ ATI-5261 treatment of high fat diet-fed *Apoe* knock-out mice decreased cholesterol levels in both plasma and feces and reduced atherosclerotic lesions.^289^ For CS-6253, a reduction of Aβ_42_ levels and tau protein phosphorylation in transgenic humanized *APOE4* mice was demonstrated, which was accompanied by improved cognitive functions.^293^ Interestingly, an elevation of ABCA1 protein was also observed in treated mice.^293^ Indeed, a stabilization and/or induction may also have contributed to the observed effects. However, the proven direct binding of these agents suggested that activation takes place as the major mode of action. Nonetheless, CS-6253 has not been tested in AD mouse models so far, and being a peptide, it would not be suitable for oral application in patients.

### Small-molecule regulators of ABCA transporters

The herein discussed regulators interfere with ABCA transporter expression and/or trafficking. Important representatives are depicted in Figure 2 and additional information is given in terms of their mode of modulation. Since many different pathways are involved in ABCA transporter regulation, Figure 3 provides a general overview of participating proteins and protein families in terms of the most studied ABCA transporter, ABCA1.

#### Inducers

##### ABCA1 - LXR and RXR pathways

Given the findings in AD mouse models with knock-out of *ABCA1/Abca1* or overexpression of ABCA1, upregulating ABCA1 activity may be a therapeutic strategy for decreasing Aβ pathology in AD. *ABCA1* is under the transcriptional control of the nuclear receptors liver-X-receptor (LXR) and retinoid-X-receptor (RXR),^294-296^ which can be targeted by small-molecule agonists of LXR and RXR to induce ABCA1 expression (Figure 3). Numerous studies reported that treatment of *APP*-transgenic mice with LXR or RXR agonists decreased Aβ load^126,297-301^ and/or improved cognitive impairment.^126,297,298,300^ Other studies reported cognitive improvement without significant changes in Aβ load in *APP*-transgenic mice treated with LXR agonists.^302,303^ LXR and RXR agonists have already been described extensively as potential therapeutics in the literature, also with respect to AD.^304^ The present review will focus on those agonists that were reported in clear association with ABCA1.

###### Oxysterols and retinoic acids

22-(*R*)-hydroxycholesterol (Figure 2) has been established as the natural gold standard for *ABCA1/Abca1* induction through LXR activation,^122,205,249,252,259,262-264,268,277,278,305-315^ while 9-*cis* retinoic acid (Figure 2) became the natural gold standard for RXR activation.^122,245,249,259,262,264,277,278,309,311,313,316^ The inducing effects were described both on *ABCA1/Abca1* mRNA^122,205,252,263,264,305,307-311,313,315-317^ and ABCA1 protein levels.^122,252,263,264,306,309-311,316,318^

Other oxysterols like 4-hydroxycholesterol, 20-(*S*)-hydroxycholesterol, 22-(*S*)-hydroxycholesterol, 24-hydroxycholesterol, 24-(*S*)-hydroxycholesterol, 25-hydroxycholesterol, 27-hydroxycholesterol, and cholesterol itself also induced *ABCA1*/*Abca1* mRNA^205,305,313,315,319-327^ and ABCA1 protein levels.^321,328^ The increase in ABCA1 protein was functionally confirmed by an enhanced cholesterol^305,306,313,315,318^ and phospholipid efflux,^311,318^ as well as reduced total cholesterol influx.^305^ Specifically 22-(*R*)-hydroxycholesterol and cholesterol induced both *LXRA/Lxra* and *LXRB/Lxrb*.^310,321^ Additionally, cholesterol also induced murine peroxisome proliferator-activated receptor γ (PPAR-γ) mRNA (*Pparg*),^321^ which represents an important alternative pathway for *ABCA1/Abca1* induction. Furthermore, 24-(*S*)-hydroxycholesterol reduced in parallel the sterol regulation element-binding protein 2 (SREBP2) gene expression (*Srebp2*).^323^ The SREB protein family also represents another important pathway in *ABCA1/Abca1* regulation.

The 9-*cis*-retinoic acid derivative *all-trans*-retinoic acid (ATRA) significantly increased *ABCA1*/*Abca1* mRNA and ABCA1 protein content in murine and human macrophages, which was paralleled by increased *LXRA* mRNA levels in human macrophages.^329^ This increase resulted in a subsequently enhanced cholesterol efflux from murine macrophages. ATRA is an agonist of the retinoic acid receptor (RAR),^329^ which is in close relation to the RXR receptor and a potential target of retinoic acid derivatives.

###### TO901317 and GW3965

The synthetic gold standard and most studied *ABCA1*/*Abca1* inducer in the literature is TO901317 (often referred to as ‘T0901317’; Figure 2).^205,245,250,252,259,260,262,264,271,272,279,280,282,308,310,317,319,322,324,326,328-345^ TO901317 targeted both the LXR-α^250,310,328,330,332,335,337-340,342^ and LXR-β pathways,^250,310,338,342^ which correlated to *ABCA1*/*Abca1* induction on mRNA and ABCA1 protein levels.^205,250,279,282,310,319,322,324,326,328,330-335,337-340,342,343^ In addition, an induction of *SREBP1C*/*Srebp1c* has also been observed.^336,342^ Functionally, TO901317 increased cholesterol efflux,^250,259,260,262,264,282,319,324,329,331,342^ decreased intracellular Aβ content, and increased Aβ secretion from different murine brain cells.^126,345^ Further, it reduced Aβ_25-35_-mediated toxicity toward cells by induction of *Abca1*.^280^ In addition, TO901317 mitigated memory deficits in high-fat diet-fed *APP23* mice, reducing both plaque and soluble Aβ protein levels.^344^ Besides, TO901317 reduced methionine-(homocysteine)-induced atherosclerotic lesions in *Apoe* knock-out C57BL/6 mice.^335^ These findings were paralleled by an increase of *Abca1* mRNA and ABCA1 protein content,^335^ suggesting a potential relevance of TO901217 in AD therapy, although it must be taken into account that LXR activators, in particular TO901317, were demonstrated to have severe side effects in mice, such as neutropenia, hypertriacylglycerolemia, hepatic triacylglycerol accumulation, and hepatic steatosis.^271,346,347^

The second most common synthetic LXR-α and LXR-β agonist is GW3965 (Figure 2).^255,272,317,319,321,334,348-352^ GW3965 increased mRNA^317,319,321,348,349,351,352^ and protein levels^255,272,351^ in different ABCA1-expressing cells. Functionally, increased *Abca1* mRNA and ABCA1 protein levels correlated with enhanced cholesterol efflux.^255,351^ Strikingly, exposure of murine BV2 microglia to GW3965 reduced Aβ_42_ levels due to an enhanced degradation of Aβ,_42_^126^ suggesting that ABCA1 contributes to general Aβ degradation. Finally, GW3965 significantly increased *Abca1* transcription in C57BL/6 mice,^334,351^ and improved contextual memory as well as Aβ pathology in TG2576 mice,^126^ emphasizing its high relevance in AD therapy.

##### ABCA1 - other LXR agonists and inducers

###### Sterane and sterane-like natural compounds

Several sterane derivatives were demonstrated to target LXR-α and LXR-β activation^253,307,310,353^ and/or LXRa/Lxra and LXRB/Lxrb upregulation,^330,332,354,355,356,357^ resulting in induction of *ABCA1*/*Abca1*. Celastrol,^330,332^ digoxin,^253^ fucosterol,^308^ certain gypenosides,^354^ ouabain,^253^ platycodin D,^355^ saikosaponin A,^356^ 24-(*S*)-saringosterol,^307^ 24-(*S*)-stigmast-5-ene-3β,24-diol,^307^ taxarasterol,^353^ testosterone,^357^ and TR1^310^ increased *ABCA1*/*Abca1* mRNA^307,308,310,330,332,353,354,356,357^ and/or ABCA1 protein content^310,253,353,354,355,357^ leading to an enhanced efflux of cholesterol *in vitro*^253,308,330,332^ and decreased intracellular cholesterol and/or phospholipid levels *in vitro*^330,332,354,356,357^ and *in vivo* in mice.^253^ The effect of fucosterol was comparable to that of the standard *ABCA1*/*Abca1* inducer TO901317.^308^ A correlation to *SREBP1(C)* upregulation^308,307,357^ and SREBP1 protein expression^357^ could be determined in case of fucosterol,^308^ 24-(*S*)-saringosterol,^307^ 24-(*S*)-stigmast-5-ene-3β,24-diol,^307^ and testosterone.^357^ In case of celastrol, the regulation of intracellular cholesterol was pinned to an activation of autophagy^330,332^ and lipophagy,^330^ which are processes that may be associated with Aβ degradation.

###### Flavonoids

The flavonoids naringenin,^339^ quercetin,^358^ and vitexin^359^ increased *ABCA1*/*Abca1* mRNA^339,359^ and ABCA1 protein levels^339,360,358^ by induction of LXRA/*Lxra* mRNA^358,359^ and LXR-α protein.^339,360^ The effect of naringenin and the standard *ABCA1*/*Abca1* inducer TO901317 were additive. Naringenin was shown to be dependent on the cAMP-activated protein kinase (AMPK) regulation (*AMPK*), as well as *SREBP1C* regulation.^339^ The AMPK pathway is another very important regulator of ABCA1 expression. Functionally, cholesterol efflux from human^339,360^ and murine^360^ macrophages was increased in the presence of naringenin.^339,360^ *In vivo*, naringenin and quercetin induced *Abca1*^360^ and ABCA1,^361,362^ as well as ABCA1-mediated cholesterol transport,^360^ which was reflected in reduced atherosclerotic lesions in the aorta of high-fat diet-fed C57BL/6 mice.^360^ In terms of quercetin, a protein increase of LXR-α and PPAR-γ was observed.^361^

Chalcones, the precursors of flavonoid biosynthesis, were also demonstrated to intervene with *ABCA1* expression. The chalcone derivatives 1h,^363^ 1m,^363,364^ and 1m-6^364^ were demonstrated to increase *ABCA1* mRNA and ABCA1 protein levels in THP-1 macrophages,^363,364^ which was accompanied by an increase in *LXRA* mRNA and LXR-α protein levels.^363^ The intracellular lipid content was decreased, while the cholesterol efflux was increased after exposure of THP1-cells to 1m-6.^364^ In addition, *SREBP1* mRNA was increased by 1m-6,^364^ and aortic atherosclerotic plaques were reduced in *Ldlr* knock-out C57BL/6 mice.^364^

###### Polyphenols and diterpenoid natural compounds

The polyphenols kuwanon G,^365^ paeonol,^252^ the *Celtis biondii*-derived compound ethyl 2,4,6-trihydroxybenzoate,^342^ and the diterpenoid farnesin^366^ increased *ABCA1*/*Abca1* mRNA^252,342,365,366^ and ABCA1 protein^252,342,365,366^ content in an LXR-α-^252,366^ and LXR-β-dependent^342^ manner, which in parallel reduced cholesterol content^252^ and increased ABCA1-mediated cholesterol efflux in various cell lines.^252,342,366^ *In vivo*, farnesin increased ABCA1 protein content and cholesterol efflux in *Apoe* knock-out C57BL/6 mice in primary peritoneal macrophages and the aorta, which was reflected in reduced atherosclerotic plaques.^366^

###### Other natural compounds

Several other natural compounds induced *ABCA1*/*Abca1* targeting LXR-α and LXR-β activation^256,272,256,349,367^ and/or *LXRA*/*Lxra* and *LXRB*/*Lxrb* induction.^331,348,350,368,369,370,371,372,373,374^ The garlic ingredient allicin,^350^ the alkaloid berberine,^256^ the coumarin bergapten A,^368^ certain *Pestalotiopsis neglecta*-derived chromene derivatives,^348^ the *Rheum palmatum*-derived anthraquinone danthron,^369^ the lacton 1,6-*O,O*-diacetylbritannilactone,^371^ epigallocatechin gallate (EGCG),^370^ the glycoside geniposide,^375^ the vegetable ingredient phenethyl isothiocyanate,^373^ the carotenoid lycopene,^372^ the *Pestalotiopsis neglecta*-derived hydroquinone pestalotioquinoside C,^349^ the alkaloid rutaecarpine,^367^ selenium,^374^ the macrolactone soraphene A,^272^ and vitamin D_3_^331^ led to increased *ABCA1*/*Abca1* mRNA^256,272,331,348,369,256,367,370,372,373^ and ABCA1 protein^256,272,331,349,350,368,369,256,367,371,373,374^ content *in vitro*^331,349,350,369,375,374^ and *in vivo*,^368,369,370,371,372,373^ enhancing cellular cholesterol efflux^256,272,256,367,369^ and reducing intracellular cholesterol content.^331,350,369,256,367,375,372,374^ Danthron also increased AMPK protein levels,^369^ while EGCG downregulated *Srebp1* mRNA and SREBP1 protein content.^370^ Lycopene induced *Ppara* mRNA in tobacco carcinogen- and cigarette smoke-exposed ferrets,^372^ while isothiocyanate induced *Pparg* mRNA as well as PPAR-γ protein content in high fat diet-fed C57BL/6 mice.^373^ The inducing effects on ABCA1 expression of vitamin D_3_ and TO901317 were additive.^331^ Danthron, EGCG, geniposide, and rutaecarpine demonstrated also reduced atherosclerotic lesions in *Apoe* knock-out C57BL/6 mice,^369,370,375,367^ and isothiocyanate ameliorated the aortic injury of the high-fat diet in the same mice.^373^

###### Pharmacological drugs

Several pharmacological drugs also demonstrated an induction of *ABCA1*/*Abca1* through LXR-α and/or LXR-β, including the a_1_-blocker doxazosin,^376^ the 5-HT_3_ receptor antagonist ondansetron,^279^ and the anesthetic propofol.^377^ Consequently, increased *Abca1* mRNA^279,376^ and ABCA1 protein^279,376^ levels were observed in human^279,377^ and murine^279,376^ macrophages^376,377^ as well as astrocytes.^279^ Functionally, ondansetron induced APOE efflux,^279^ while propofol led to increased cholesterol efflux.^377^ In addition, propofol increased *PPARG* mRNA and PPAR-γ protein content in human macrophages.^377^

Furthermore, certain antineoplastic agents interfered with ABCA1 expression *via* LXR-α and/or LXR-β. Doxorubicin demonstrated an *Lxr* activation with subsequent induction of *Abca1* mRNA and ABCA1 protein *in vitro* and *in vivo*.^250^ Functionally, doxorubicin elevated cholesterol export *in vitro*. It was shown that intra- and extracellular levels of cholesterol, cholesterol precursors, and several oxysterols were elevated after exposure to doxorubicin. These precursors included lathosterol, lanosterol, and desmosterol, while the oxysterols included 7-α-hydroxycholesterol, 7-β-hydroxycholesterol, 7-ketocholesterol, 24-hydroxycholesterol, and 27-hydroxycholesterol. The authors suggested that doxorubicin exposure induced cholesterol metabolism subsequently leading to an induction of ABCA1. Besides, idarubicin augmented also *Abca1* mRNA levels *in vitro*.

###### Synthetic compounds and HTS hits

Other synthetic compounds have been shown to induce *ABCA1*/*Abca1* expression by LXR-α and/or LXR-β induction. The polymer pyrrole-imidazole-polyamide activated a promoter region for *Abca1* expression and thereby increased cholesterol and lipid efflux from RAW264.7 cells.^376^ The authors confirmed their findings *in vivo*, revealing increased *Abca1* mRNA and ABCA1 protein content in peripheral blood mononuclear cells and the liver in C57BL/6 mice after exposure to pyrrole-imidazole-polyamide.

In addition, the LXR agonist LXR623 induced *ABCA1* mRNA and ABCA1 protein levels in two human renal adenocarcinoma cell lines^334^ as well as *Abca1* mRNA levels *in vivo* in C57BL/6 mice.^378^ This induction was reflected in reduced intracellular cholesterol and triglyceride levels.

It must be noted that several other synthetic LXR-α and LXR-β agonists induced *Abca1* expression *in vivo:* AZ1–AZ9, AZ876, BMS-852927, F1, WAY254011.^378^ Finally, an HTS approach discovered two LXR-α and LXR-β agonists as novel small-molecule *ABCA1*/*Abca1* inducers: F4 and M2.^319^

###### Synthetic approaches

A few synthetic approaches have aimed toward the development of *ABCA1*/*Abca1* inducers.^271,336,352,379-382^ The cholic acid analog 14b,^336^ the thiophene derivative CL2-57,^271^ as well as derivatives of N-benzothiazolyl-2-benzenesulfonamide,^379^ ginsenoside,^352^ and rutaecarpine,^367^ all induced *ABCA1*/*Abca1* mRNA^336,352,381^ and ABCA1 protein^271,336,379,381^ content *in vitro*^271,336,379^ and *in vivo*,^271^ targeting the LXR-α/LXR-β pathway^352^ by activation^271^ or induction^336^ of LXR-α/*LXRA*/*Lxra* and/or LXR-β/*LXRB*/*Lxrb*. *In vitro*, cholesterol efflux increased^379,381^ and intracellular cholesterol as well as lipid content were reduced,^336,352^ while plasma and liver triglycerides levels were reduced *in vivo* in high fat diet-fed C57BL/6 mice.^271^ Interestingly, 14b induced farnesoid-X-receptor (FXR) transcription (*Fxr*),^336^ and CL2-57 inhibited RXR-β, PPAR-γ, and PPAR-δ,^271^

Finally, Singh *et al.* described highly potent LXR-α and LXR-β agonists with effect at concentrations in the nanomolar range.^382^ The described podocarpic acid derivatives have not yet been demonstrated to induce *ABCA1*. However, these compounds were designated as potential ABCA1 inducers by the authors,^382^ and their high potency makes them interesting candidates for further evaluation.

Such synthetic approaches should be highlighted,^271,336,352,379-382^ as chemical derivatization of *ABCA1* inducers and elucidation of their structure-activity relationships (SAR) have not yet been comprehensively assessed. More reports are needed to gain innovative molecules that can be considered clinically for the treatment of various ABCA1-related diseases.

##### ABCA1 - other RXR agonists and inducers

In terms of synthetic RXR agonists, the 4-chromanon derivatives SPF1 and SPF2 increased Abcb1 mRNA and ABCA1 protein levels and lowered Aβ_25–35_-mediated cell toxicity *in vitro*.^280^ The same effect was observed for the RXR agonist bexarotene,^280^ an FDA approved drug against T-cell lymphoma-related cutaneous malformations. Bexarotene was used as a standard inducer of *ABCA1*/*Abca1* via the RXR pathway in several studies.^271,272,280,319,380^ Induction of *Abca1* mRNA and ABCA1 protein levels was maximal for bexarotene in combination with TO901317.^280^ Bexarotene is of particular practical relevance as a potential treatment against AD due to its *in vivo* effects. In different AD mouse models, bexarotene increased *Abca1* mRNA and ABCA1 protein levels, but also reduced cerebral load of Aβ and hyperphosphorylated protein tau, which is also a histological marker in AD and other dementias.^297,383^ This prospect led to synthetic bexarotene derivatives, specifically Z10 and Z36.^380^ Both candidates induced ABCA1 protein expression by RXR-α activation and reduced Aβ burden in the hippocampus of female *APP*/PS1 mice. This coincided with an enhanced ABCA1 protein expression in BV2 cells.

Moreover, the pan-RAR agonist TTNPB also increased ABCA1 protein content in murine macrophages in an RXR-α-dependent manner. However, the effect was generally smaller compared to the effect of ATRA.^329^ Finally, a combination of the LXR and RXR agonists RO0721957 and RO0264456 increased *ABCA1* mRNA in THP-1 macrophages accompanied by increased cholesterol efflux.^384^ RO0264456 was demonstrated to increase ABCA1 protein content in combination with TO901317.^260^

##### ABCA1 – protein kinase C (PKC), AMPK, and p38 mitogen-activated protein kinase (MAPK)

An alternative approach to induce *ABCA1* is targeting the PKC pathway (Figure 3). PKC agonists were extensively used to induce *ABCA1*/*Abca1* mRNA and ABCA1 protein levels.^230,248,249,255,265,266,273,278,289-292,384-387^ Prominent PKC agonists include cAMP^313^ as well as synthetic derivatives, such as 8-Bromo-cAMP (8-Br-cAMP; Figure 2),^230,249,255,266,290,292^ 8-(4-chlorophenylthio)-cAMP (CPT-cAMP),^273,291,384^ and dibutyryl-cAMP.^385-387^ The observed effects ranged in the same order of magnitude as the combination of 22-(*R*)-hydroxy-cholesterol and 9-*cis*-retoic acid.^313^ The increase in *ABCA1*/*Abca1* mRNA and ABCA1 protein levels was reflected in an enhancement of ABCA1-mediated cholesterol and phospholipid efflux,^249,255,386^ and increased APOA1 binding to murine RAW264.7 macrophages.^385-387^ Similar observations have been made for the PKC stimulant phorbol 12-myristate 13-acetate (PMA), which induced ABCA1 protein expression and ABCA1-mediated cholesterol and phospholipid release.^386^ PMA is also the standard substance used to differentiate human monocytic leukemia cells into THP-1 macrophages – a standard host system for ABCA transporter evaluation.^231,245,249,256,268,272,275,292,308,310,312-316,321,328,335,338,339,341,342,360,363,364,366,377,384,388-397^

Regarding the AMPK pathway (Figure 3), the natural compound curcumin induced *ABCA1*/*Abca1* mRNA^338,388^ and ABCA1 protein levels^388,394^ as well as cholesterol efflux^338,388,394^ in THP-1^338,388,394^ and RAW264.7^394^ macrophages, which was also mediated through LXR-α activation.^338^ However, these LXR-α activating effects were much more pronounced in combination with the gold standard TO901317.^338^ Other AMPK-targeting agents are A-769662 and metformin,^398^ which induced *ABCA1*/*Abca1*,^398^ *LXRA*/*Lxra*,^396,398^ and *LXRB*/*Lxrb*^396,398^ in human^398^ and murine (primary) macrophages,^398^ leading to increased cholesterol efflux.^396^

Concerning the MAPK pathway (Figure 3), the sterane glycoside ginsenoside compound K increased *Abca1* mRNA and ABCA1 protein levels in murine macrophages, reducing intracellular lipid content and promoting autophagy.^399^ These effects were pinned to a negative impact on the MAPK pathway. Finally, a synthetic inhibitor of MAPK, SB203580, was shown to induce ABCA1 protein in combination with the above mentioned geniposide *in vitro* in murine macrophages.^375^

##### ABCA1 - the PPAR Pathway

Another well-known approach to induce ABCA1 involves the PPAR pathway (Figure 3).^268,272,295,309,315,321,326,327,337,343,395,400-409^ Certain *PPAR/Ppar* inducers and/or PPAR activators have been described above, as these modulators also have effects on the LXR pathway.^321,361,372,373,377^

Several natural compounds target the PPAR pathway, such as the flavonoids homoeriodictyol,^402^ hesperetin-7-*O*-β-_d_-glucopyranoside,^402^ scutellarein,^403^ and the antimycotic trichostatin A.^410^ These compounds increased *Abca1*^402^ and *Pparg*^402^ mRNA as well as ABCA1,^402,410^ PPAR-α,^403^ and PPAR-γ^402,410^ protein levels *in vitro*^402,410^ and *in vivo*.^403^ Decreased intracellular cholesterol levels were also observed.^402^ Trichostatin A reduced aortic atherosclerotic plaques in high-fat diet-fed *Apoe* knock-out mice,^410^ and an upregulation of ABCA1, PPAR-γ, and LXR-α/β protein levels was observed in aortic cells as well as peritoneal macrophages.^410^

Several drugs and drug-like PPAR agonists were revealed to induce *ABCA1*/*Abca1* mRNA and/or ABCA1 protein content, including the PPAR-α agonists fenofibrate,^326,400,404^ pemafibrate (K-877),^405^ Wy14643,^268,343^ and RPR-5,^268^ as well as the PPAR-γ agonists efatutazone,^337^ pioglitazone,^272,309,326,395,407^ pitavastatin,^343^ prostaglandin J2 (PG-J2),^268,327^ rosiglitazone (Figure 2),^268,309,315,408,409^ troglitazone,^268^ and GW7845,^315^ but also the broad-spectrum PPAR-α, PPAR-β, and PPAR-γ agonist bezafibrate^268,327^ and the multitarget PPAR-α, PPAR-γ, and PPAR-δ agonist tetradecylthioacetic acid.^401^ This induction was observed for *ABCA1*/*Abca1* mRNA^268,315,343,401,405^ as well as ABCA1 protein levels,^268,337,343,395,405,409^ and was functionally confirmed by increased cholesterol efflux.^268,315^ A connection between the PPAR and LXR pathways has also been drawn,^268,326,327,337,400^ highlighting the importance of both pathways for *ABCA1*/*Abca1* induction. Furthermore, fenofibrate had a positive impact on both the LXR-α and AMPK pathways^400^ Certain PPAR agonists have been used as standard inducers of *Abca1*, *e.g.*, pioglitazone^407^ and rosiglitazone.^408^

Synthetic PPAR agonists were also reported to induce ABCA1.^406^ The benzothiazole derivative E3317 dose-dependently increased *ABCA1*/*Abca1* mRNA and ABCA1 protein levels though PPAR-γ activation in several cell lines.^406^ This was reflected in decreased cholesterol efflux and reduced intracellular cholesterol content. Finally, a molecular docking approach to discover novel PPAR agonists has yielded GQ-11, which induced *Abca1* mRNA in livers of C57BL/6 *Ldlr* knock-out mice.^407^

##### ABCA1 - the 3-hydroxyl-3-methyl glutaryl-(HMG)-CoA-reductase pathway

Other targets for *ABCA1*/*Abca1* induction are the 3-hydroxyl-3-methylglutaryl-(HMG)-CoA-reductase and cellular cholesterol synthesis (Figure 3).^318,343^ Several HMG-CoA-reductase inhibitors such as atorvastatin (Figure 2),^330,343,362^ fluvastatin,^312,411^ mevastatin (compactin),^318^ pitavastatin,^318,343^ and simvastatin^312,343^ increased *ABCA1*/*Abca1* mRNA^312,343^ and ABCA1 protein levels,^362,411^ as well as ABCA1-mediated cholesterol efflux.^318^ These data are surprising, as one might expect the loss-of-function of an enzyme in the cholesterol synthesis pathway to induce a decrease of ABCA1, preventing cholesterol depletion from cells.^314,384,412^ Conversely, the overproduction of cholesterol leads to the opposite effect, as demonstrated for mevalonate, which is a building block of cholesterol synthesis^413^ and has been demonstrated to increase *ABCA1*/*Abca1* mRNA^312,314^ and to abrogate *Abca1* downregulation.^312^ Pitavastatin addressed SREBP-driven promotor regions upregulating *Abca1* mRNA levels,^343^ and atorvastatin reduced atherosclerotic plaques in *Apoe* knocked-out C57BL/6 mice by induction of ABCA1 protein content in the murine aorta.^362^

##### Other ABCA1 inducers

###### Sterane and sterane-like natural compounds

Several other agents were reported to induce *ABCA1*/*Abca1* mRNA and/or ABCA1 protein level(s), with some studies reporting a unique mechanism of action for these agents. Such compounds include the sterane derivative ponasterone A (ecdysone; ABCA1 protein; ABCA1-mediated cholesterol and phospholipid transport),^202^ and the enoxolone derivative glycyrrhizine (ABCA1 protein).^414^ In addition, the sterane derivative and farnesoid-X-receptor (FXR) activator obeticholic acid induced *Abca1* mRNA levels *in vitro* in the ileum of *Srb1*-deficient C57BL/6 mice.^415^ In THP-1 macrophages, the sterane-like maslinic acid induced *ABCA1* mRNA levels, paralleled with an increased cholesterol efflux from these cells.^390^ Finally, the *Salvia miltiorrhiza*-derived tanshindiol C was demonstrated to induce peroxiredoxin 1 mRNA (*Prdx1*) and protein (PRDX1) content in murine RAW264.7 cells.^416^ *Prdx1* was demonstrated to regulate *Abca1* mRNA and ABCA1 protein expression. A reduction of intracellular cholesterol levels in murine peritoneal macrophages could also be observed.

###### Flavonoids

The flavonoids daidzein (Figure 2),^309^ kaempferol,^397^ and pratensein^309^ induced *ABCA1* mRNA^309,397^ and ABCA1 protein levels^309^ as well as ABCA1-mediated cholesterol efflux.^397^ In addition, hesperetin-7-*O*-rutinosid (hesperidin) abrogated the negative effect of varenicline on ABCA1 protein expression in RAW264.7 macrophages.^417^ The authors could underpin their findings with a reduction of aortic atherosclerotic plaques in *Apoe* knock-out C57BL/6 mice along with reduced lipid levels in peritoneal macrophages derived from these mice.

###### Polyphenols and polyphenol-like natural compounds

Several polyphenols and polyphenol-like compounds induced *Abca1* mRNA^408,418^ and ABCA1 protein^393,404^ levels in murine^393,404,408,418^ and human^393^ macrophages, leading to an increased cholesterol efflux.^404,408,418^ These include certain *Cannabis sativa*-derived stilbenoids^404^ as well as the *Tadehagi triquetrum*-derived phenylpropanoid glycosides urolithin A^418^ and urolithin B (sulfate).^393^ *In vivo*, atherosclerotic plaques were reduced after urolothin B treatment. One phenylpropanoid glycoside was demonstrated to increase *Lxra*, but none of the other compounds could confirm these results. Given that the effect of all compounds on ABCA1 expression was similar, it is likely that another, yet unknown pathway was the major contributor to the observed effects.

###### Other natural compounds

Sodium butyrate induced *Abca1* mRNA and ABCA1 protein levels in murine RAW264.7 cells, accompanied by an increased efflux of cholesterol from these cells.^419^ This induction was reflected by increased ABCA1 protein content *in vivo*, reduced plasma cholesterol and triglyceride levels, and reduced aortic atherosclerotic lesions and hepatic steatosis in high fat diet-fed *Apoe* knock-out C57BL/6 mice.

###### Pharmacological drugs

Several pharmacological drugs induced *ABCB1*/*Abca1* mRNA^309,420,421^ and ABCA1 protein,^309,391,421^ including the anti-obesity drug orlistat,^391^ the antibiotic sulfoxaflor,^420^ the leukotriene receptor antagonist zafirlukast,^421^ as well as the anthracyclines aclarubicin^309^ and pyrromycin.^309^ Zafirlukast in particular reduced intracellular cholesterol and lipid content in oxidized LDL-(oxLDL)-induced lipid-overloaded RAW264.7 macrophages, and increased cholesterol efflux from these cells.^421^

Finally, it should be highlighted that mifepristone has frequently been used in a mifepristone-inducible transfection system to stabilize and increase *ABCA1* expression in *ABCA1*-transfected baby hamster kidney (BHK)-21 cells. This *ABCA1* induction could be functionally confirmed by increased ABCA1-mediated cholesterol and phospholipid efflux.^245,273,422^

###### Synthetic compounds, HTS hits, and synthetic approaches

The purinergic P2Y7 receptor antagonists AZ-1, AZ-2, and AZ10606120 increased *ABCA1* mRNA and ABCA1 protein levels and resulted in enhanced cholesterol efflux from human CCFSTTG1 astrocytoma cells.^423^ The polychlorinated biphenyl quinone 2,3,5-trichloro-6-phenyl-[1,4]-benzoquinone (PCB29-pQ)^424^ and the fluorescigenic pyrazoline derivative 5 (FPD5)^425^ increased *Abca1* mRNA^424^ and ABCA1 protein^425^ content in RAW264.7 macrophages and reduced cholesterol content in these cells.^424,425^ *In vivo*, FPD5 reduced aortic lipid and cholesterol content and atherosclerotic lesions in *Apoe* knock-out C57BL/6 mice.

##### Inducers of other ABCA transporters

###### ABCA2 and ABCA3

As detailed above, ABCA2 and ABCA3 are believed to contribute to multidrug resistance in cancer.^171,232,239,241,242^ In human K562 leukemia cells, it was demonstrated that the tyrosine kinase inhibitor (TKI) imatinib induced increased levels of *ABCA2* mRNA and ABCA2 protein.^236^ Furthermore, the TKIs dasatinib, imatinib, and nilotinib increased *ABCA3* mRNA levels in various cancer cell lines as well as in TKI-treated leukemia patients.^426^ The antimetabolite 5-fluorouracil (5-FU) induced expression of *ABCA3* mRNA in a cholangiocarcinoma cell line,^427^ and methotrexate increased *ABCA2* and *ABCA3* mRNA in a leukemia cell line.^242^ Finally, the steroid hormone progesterone,^179^ the antibiotic sulfoxaflor,^420^ and the endosomal cholesterol transport inhibitor U18666A^179^ induced *ABCA2*/*Abca2* transcripts^420^ in *Aphis gossypii*^420^ as well as in *ABCA2*-transfected Chinese hamster ovary (CHO) cells and HepG2 cells^179^

###### ABCA5 and ABCA6

As discussed earlier, cholesterol and its derivatives have been shown to induce *ABCA1*/*Abca1* mRNA and/or ABCA1 protein levels.^122,205,249,252,259,262-264,268,277,278,305-315,319-328^ Induction by cholesterol has also been demonstrated for *Abca5* mRNA and ABCA5 protein levels in RAW264.7 macrophages.^321^ This effect relied on the induction of *Lxra, Lxrb*, and *Pparg*. Consequently, several LXR and PPAR agonists increased *Abca5* expression, including bezafibrate (PPAR-α, PPAR-β, and PPAR-γ; *Abca5* mRNA and ABCA5 protein), GW3965 (LXR; *Abca5* mRNA), rosiglitazone (PPAR-γ; *Abca5* mRNA), and troglitazone (PPAR-γ; *Abca5* mRNA) in murine RAW264.7 macrophages.^321^ In addition, the HMG-CoA-reductase inhibitor atorvastatin increased *Abca5* mRNA and ABCA5 protein levels.^321^ Interestingly, the ABCA1 inhibitor tacrolimus^245^ showed induction of *ABCA5* mRNA in human brain microvascular endothelial cells.^428^

The HMG-CoA-reductase inhibitors lovastatin and mevastatin resulted in an induction of *ABCA6* mRNA in the human endothelial cell line EA.hy926.^429^ Finally, in an *Abca12* pig model of the rare and lethal skin disease Harlequin ichthyosis, it was demonstrated that treatment with the synthetic retinoid acitretin leads to a compensatory induction of *Abca6* mRNA.^430^

###### ABCA7

Similarly to ABCA1,^202^ the sterane derivative ponasterone A increased both ABCA7 protein expression and ABCA7-mediated transport, mainly of phospholipids, but also of cholesterol to a small extent.^202^

HMG-CoA-reductase inhibitors were described above to interfere with *ABCA1*/*Abca1*^312,318,330,343,362,411^ and *Abca5*^321^ expression. In addition, certain compounds were also demonstrated to interfere with *Abca7* expression.^205,431^ These include pravastatin^205,431^ and rosuvastatin (Figure 2).^431^ These agents increased *Abca7* mRNA and ABCA7 protein levels *in vitro*,^205,431^ whilst pravastatin had the same effects *in vivo* in murine peritoneal macrophages.^431^ Surprisingly, this increase of *Abca7* mRNA and ABCA7 protein levels was accompanied by a downregulation of *Lxra* and upregulation of *Srebp2 in vitro*.^431^ Functionally, pravastatin and rosuvastatin reduced intracellular cholesterol content^431^ and induced phagocytosis *in vitro* and *in vivo*.^431^ These effects occurred in response to an ABCA1 downregulation by HMG-CoA-reductase inhibitors as described earlier.^312,321,384,432,439,429^ Due to their functional similarity, the upregulation of ABCA7 could be a compensatory mechanism to counteract the loss of ABCA1.^198^ Similarly, the observed *Lxra* down- and *Srebp* up-regulation may be a compensatory mechanism to counteract the loss of intracellular cholesterol.

Finally, as described for ABCA1,^422^ exposure of *ABCA7*-transfected BHK-21 cells to mifepristone increased ABCA7 protein content and ABCA7-mediated transport of phospholipids and, to a much lesser extent, of cholesterol.^422^

###### ABCA8

*ABCA8* mRNA and ABCA8 protein content were induced by gemcitabine in PANC-1 and CFPAC-1 human pancreatic cancer cells.^221^ In rat liver, an induction of *Abca8* was demonstrated *via* microarray analysis of cDNA when the rats were exposed to polyethyleneglycol-block-polylactide nanoparticles.^433^

###### ABCA12

Several LXR and PPAR agonists induced ABCA12/Abca12 expression, such as 22-(R)-hydroxycholesterol (LXR),^434^ TO901317 (LXR),^430,434^ ciglitazone (PPAR-γ),^434^ GI 251929X (PPAR-γ),^434^ troglitazone (PPAR-γ),^434^ ceramide N-hexanoyl-D-erythro-sphingosine (PPAR-δ),^435^ and GW610742 (PPAR-δ).^434^

Interestingly, inhibition of certain enzymes to prevent ceramide processing elevated intracellular ceramide content and subsequently *ABCA12* mRNA levels.^435^ These enzymes include, for example, the glycosyl-ceramide-transferase synthase [_d_-threo-1-phenyl-2-hexadecanoylamino-3-morpholino-l-propanol (_d_-PPMP), _d_-threo-1-phenyl-2-palmitoyl-3-pyrrolidinopropanol (_d_-PPPP / P4) and _dl_-threo-1-phenyl-2-de-canoylamino-3-morpholino-1-propanol (_d_-DDMP)], the sphingomyelin synthase [tricyclo[5.2.1.0^2,6^]decanyl)ethanedithioic acid (D609 xanthate)], as well as the ceramidase [D-erythro-2-tetradecanoylamino-1-phenyl-1-propanol (_d_-MAPP) and (_d_-NMAPPD / B13)].^435^

#### Downregulators

##### ABCA1

###### LXR and RXR pathways – intrinsic substrates

The intrinsic metabolite asymmetric dimethylarginine (ADMA) reduced *Abca1* mRNA and ABCA1 protein levels in human and murine J744 macrophages in combination with oxLDL, resulting in increased intracellular cholesterol and triglyceride levels.^392^ This was accompanied by decreased efflux of cholesterol from these cells. The authors suggested a negative effect on the LXR-α pathway. In this regard, the LXR-α downregulator homocysteine significantly reduced *ABCA1*/*Abca1* mRNA and ABCA1 protein expression *in vitro* in THP-1 macrophages as well as *in vivo* in macrophages from *Apoe* knock-out C57BL/6 mice.^335^ The cattle metabolite dipeptide phenylalanine-proline decreased *ABCA1* mRNA and ABCA1 protein levels in human colorectal adenocarcinoma-derived CaCo-2 cells.^436^ The observed downregulation of *LXRB* mRNA could explain the negative impact on ABCA1 expression. *In vivo*, the jejunal *Abca1* mRNA levels were decreased in Wistar rats.^436^

The ABCA1 substrate α-tocopherol^230^ reduced *ABCA1*/*Abca1* mRNA levels *in vitro* and *in vivo*.^231^ The same effects were observed for γ-tocopherol *in vitro*, most likely through the same mechanism. The authors suggested a negative impact on the LXR pathway due to deprived oxycholesterol derivatives after α-tocopherol treatment both *in vitro* in Hep3B cells and *in vivo* in rat liver.^231^

###### LXR and RXR pathways - sterane and sterane-like natural compounds

Cholesterol and its derivatives have extensively been used to induce *ABCA1*/*Abca1* expression^122,205,249,252,259,262-264,268,277,278,305-315,319-328^ However, mid-term exposure to excess cholesterol decreased ABCA1 expression though a negative impact on *Lxra, Lxrb*, and *Pparg expression*.^321^ Similar observations have been made for the sterol derivative dexamethasone, which also reduced *ABCA1*/*Abca1* mRNA and ABCA1 protein expression *in vitro* and *in vivo* by downregulation of *LXRA*/*Lxra* mRNA and LXR-α protein levels as well as upregulation of *Srebp2* and HMG-CoA-reductase gene expression (*Hmgcr*).^437^ Finally, an *Abca1* mRNA reduction was observed in murine RAW264.7 macrophages for the *Thelenota ananas*-derived saponin desulfated holothurin A.^438^ Interestingly, *Hmgcr* was downregulated after exposure to desulfated holothurin A, which contradicts other findings.^437^

###### LXR and RXR pathways – other natural compounds

Certain chalcone derivatives also *caused* reduced expression of ABCA1 protein.^363^ In addition, lipopolysaccharides reduced ABCA1 protein content in endometrial endothelial cells from C57BL/6 mice, which was accompanied by increased cholesterol levels in these cells.^374^ A parallel reduction in LXR-α protein was also observed. Finally, the carcinogenic agent *N*-nitrosodiethylamine (NDEA) demonstrated *in vivo* in Wistar albino rats a downregulation of *Lxra* and *Lxrb* mRNA as well as LXR-α and LXR-β protein levels and, subsequently, ABCA1 protein.^368^

###### LXR and RXR pathways – synthetic compounds and HTS hits

In terms of other LXR antagonists and downregulators, GSK2033 (Figure 2),^272,330,333^ 5CPPSS-50, ^357^ and SR9243^333^ reduced *ABCA1* mRNA and ABCA1 protein expression.^272,330,333,357^

###### HMG-CoA-reductase pathways – intrinsic substrates and pharmacological drugs

The peptide hormone angiotensin II reduced cholesterol efflux from murine peritoneal macrophages.^439^ This reduction could be reversed by the angiotensin II receptor antagonist losartan. The authors concluded that ABCA1 was not involved in this process, as no concurrent change in *Abca1* expression was observed.^439^ However, in another report, angiotensin II indeed demonstrated a reduction of *ABCA1* mRNA and ABCA1 protein levels in human podocytes.^440^ The authors concluded a contribution of the HMG-CoA-reductase, SREBP1, and SREBP2.^440^

Geranylgeraniol pyrophosphate (GGPP; Figure 2), a product of the mevalonate pathway, reduced *ABCA1* mRNA expression in human macrophages, which was blocked by the prenylation inhibitors L836,978 and L-839,867.^314^ In addition, a reduction of ABCA1-mediated cholesterol export was observed, which is also true for mevalonate itself.^318^ GGPP was used as a standard *ABCA1* downregulator in certain studies.^279,354,366^

As discussed above, atorvastatin,^343^ fluvastatin,^312^ pitavastatin,^318,343^ and simvastatin^312,343^ have been shown to increase *ABCA1*/*Abca1* mRNA levels,^312,343^ and to enhance ABCA1-mediated cholesterol efflux.^318^ However, atorvastatin,^312,321,384^ fluvastatin,^312^ pitavastatin^432^ and simvastatin^312,384^ have also been reported to reduce ABCA1/*Abca1* transcription^312,321,384,432^ and ABCA1-mediated cholesterol efflux.^384,431^ These observations are in agreement with other reports on HMG-CoA-reductase inhibitors that downregulated ABCA1.^312,431^ In particular, lovastatin,^312^ mevastatin (compactin),^412^ pravastatin,^431^ and rosuvastatin^431,441^ reduced *ABCA1*/*Abca1* mRNA^312,431^ and ABCA1 protein^431^ levels. These findings are expected given that the loss of cholesterol by interruption of cholesterol synthesis leads to a compensatory reduction of cholesterol efflux.^314,384,412^ The contradictory results relating to ABCA1 may be caused by the use of variable experimental conditions between studies, such as different cell lines, assay methodologies, or small-molecule-related aspects, such as concentration, distribution, and protein binding.

Finally, a similar interconnection between HMG-CoA and ABCA1 was drawn for the antineoplastic agent mitotane, which downregulated *ABCA1* mRNA^441^ and increased intracellular cholesterol levels.^333,441^ However, mitotane in combination with LXR antagonists and *LXR* downregulators had an inverse effect on mRNA regulation, increasing ABCA1 expression.^327^

###### PKC pathway - intrinsic substrates

Interestingly, it was also demonstrated that long-term exposure to low concentrations of 8-Br-cAMP, a standard *ABCA1*/*Abca1* inducer,^230,249,255,266,290,292^ led to decreased APOE secretion from human monocyte-derived macrophages.^266^ APOE secretion can be considered as a surrogate marker for ABCA1-mediated cholesterol transport.

###### PPAR pathway – pharmacological drugs and synthetic compounds

Regarding the important PPAR pathway, it must be noted that troglitazone, indicated above as an *ABCA1* inducer,^268^ was also reported to downregulate ABCA1 transcription.^321^ These inconsistent effects may be explained partially by the different concentrations used (1 μM vs 10 μM),^268,321^ but may also be related to cross-talk between the PPAR, LXR, and mevalonate pathways. The PPAR-γ antagonist GW9662 (Figure 2) reduced ABCA1 protein levels.^406^

###### Other ABCA1 downregulators – natural compounds

Other small-molecules have been reported to act as *ABCA1*/*Abca1* downregulators, acting independently of the previously mentioned LXR, RXR, PPAR, and HMG-CoA-reductase pathways. Natural compounds such as α,β-unsaturated carbonyl derivative acrolein,^442^ the polyphenol bisphenol A,^443^ and the polyphenol 1,2,3,4,6 penta-*O*-galloyl-β-_d_-glucose^444^ demonstrated an *Abca1* mRNA^443,444^ and ABCA1 protein^442^ downregulation *in vitro*^443,442^ and *in vivo*.^444^ The effect of acrolein could be abrogated by 3-hydroxytyrosol,^442^ an inducer of ABCA1 protein content.^445^

*SREBP2* has been demonstrated to be targeted by EGCG in high fat diet-fed transgenic SREBP^+/+^ Wistar rats, resulting in *Abca1* mRNA downregulation, while an *Abca1* mRNA upregulation could be observed under the same conditions in *SREBP* knock-out Wistar rats.^446^

###### Other ABCA1 downregulators – pharmacological drugs

Exposure of the human non-small cell lung cancer lines A549 and H358 to the antiepileptic drug valproate led to downregulation of *ABCA1* mRNA and ABCA1 protein levels through a histone deacetylase 2-(HDAC2)-mediated mechanism. In parallel, the authors observed an increased sensitivity of these cells to cisplatin.^447^

The selective estrogen receptor modulators raloxifene, tamoxifen, and toremifene were reported to reduce ABCA1 protein content in THP-1 macrophages along with decreased cholesterol efflux and increased intracellular cholesterol levels.^341^ Tamoxifen and raloxifene treatment decreased serum HDL-cholesterol levels in mice. In addition, tamoxifen reduced cholesterol levels in serum, liver, and feces of mice after injection with cholesterol-loaded macrophages.^341^ Interestingly, the downregulation of ABCA1 protein content by these estrogen receptor modulators could not be demonstrated for murine liver, indicating a macrophage-specific effect.^341^

Varenicline, a drug used in smoking cessation, was shown *in vivo* to promote aortic atherosclerotic lesions in *Apoe* knock-out C57BL/6 mice.^417,448^ The authors demonstrated that intracellular lipid content in peritoneal macrophages was increased, and a decreased ABCA1 protein expression was confirmed *in vitro* in RAW264.7 macrophages. Finally, the antineoplastic agent gefitinib reduced ABCA1 protein content in various non-small cell lung cancer cell lines.^400^

###### Other ABCA1 downregulators – synthetic compounds

The plasticizer dibutyl phthalate^389^ and the PI3K/AKT inhibitor LY294002^421^ reduced *ABCA1* mRNA^389^ and ABCA1 protein^389,421^ expression and increased cellular cholesterol and lipid levels^389^ in human^389^ and murine^421^ macrophages.

The sphingosine kinase 1 and 2 inhibitor 4-{[4-(4-chlorophenyl)-2-thiazolyl]amino}phenol was demonstrated to downregulate ABCA1 protein expression in murine primary macrophages, which was dependent on the sphingosine kinase 2 as well as the sphingosine-l-phosphate receptor.^306^ This ABCA1 protein downregulation was accompanied by a reduced cholesterol efflux.

The acyl coenzyme A cholesteryl acyl transferase (ACAT) inhibitor ATR-101 reduced *ABCA1* mRNA levels and induced an increase in intracellular cholesterol content in H295R cells.^251^ The authors suggested that this was caused by inhibition of ABCA1 but provided no clear proof of direct inhibition of ABCA1. Therefore, this compound was classified as a downregulator.

##### Other ABCA transporters

###### ABCA2 and ABCA3

Compared to ABCA1, knowledge relating to downregulators of the other ABCA transporters is very limited. As discussed above, human leukemia cells exposed to imatinib displayed increased *ABCA2* mRNA and ABCA2 protein expression.^236^ Celecoxib abrogated this effect.^236^ A similar observation was reported for ABCA3, where the anti-inflammatory drug indomethacin and the ABCA1 inhibitor sirolimus^245^ (Figure 2) downregulated *ABCA3* mRNA in various cancer cell lines.^426,449,450^ This treatment also resulted in a sensitization of these cell lines toward the TKIs dasatinib, imatinib, and nilotinib when treated with indomethacin.^426^

Other compounds were also reported to downregulate *ABCA3*/*Abca3* including the flavonoid genistein,^451^ lipopolysaccharides^452^ – already demonstrated above as ABCA1 protein downregulators^374^ – and the translocator protein ligand PK11195.^453^ The effect of lipopolysaccharides could be abrogated by ascorbic acid (vitamin C).

###### ABCA5–ABCA9

Interestingly, the ABCA8 inhibitor^222^ and ABCA1 protein inducer^253^ digoxin downregulated *Abca5* and *Abca7*–9 in murine liver.^454^ The HMG-CoA-reductase inhibitors lovastatin and mevastatin downregulated *ABCA6* mRNA in human umbilical vein endothelial cells.^429^ The cholesterol derivative 25-hydroxycholesterol, which was introduced above as an *ABCA1* mRNA inducer,^327^ showed the opposite effect on *ABCA7* mRNA.^324^ This finding is in agreement with a report stating that excess cholesterol reduced ABCA7 protein content in both human and murine fibroblasts.^205^

#### Stabilizers of ABCA transporters

Stabilizers are compounds that promote functional activity of ABC transporters through increasing their presence at the site of action (*e.g.*, the cell membrane) either without interfering with mRNA or protein levels, or in addition to these effects. The categorization is difficult, as the necessary information regarding many modulators of ABCA transporters is lacking and the underlying mode of modulation cannot be precisely identified. In this section, we consider only those modulators which predominantly interfere with ABCA1 trafficking, with relatively minor or no additional modes of action/modulation. Stabilizers are of particular interest, as they may represent a novel generation of functional ABC transporter activators, expanding treatment options for several diseases, particularly AD.

##### ABCA1

Probucol and cyclosporine A were demonstrated above to decrease ABCA1 turnover and increasing ABCA1 protein content at the cell membrane.^246,275^ Arakawa *et al.* demonstrated that the probucol metabolites spiroquinone and diphenoquinone did not inhibit ABCA1-mediated transport like their parent compound but rather increased the fraction of functional ABCA1 in the cell membrane.^275^ This stabilization led to increased cholesterol and phospholipid efflux. Both effects were observed at very low nanomolar concentrations,^275^ while *Abca1* mRNA remained stable.^275^ Strikingly, spiroquinone and diphenoquinone decreased vascular lipid deposits *in vivo* in cholesterol-fed rabbits,^275^ which may be of relevance for AD and potentially other neurodegenerative diseases.

A similar mode of stabilization, albeit with less potency and no *in vivo* confirmation, has been observed for the flavonoid wogonin,^254^ the olive oil-derived compound erythrodiol,^395^ and certain thiol proteinase inhibitors, in particular *N*-acetyl-Leu-Leu-norleucinal and leupeptin.^316,386^ Finally, the *ABCA1* mRNA and ABCA1 protein inducer testosterone was demonstrated to promote ABCA1 trafficking to the cell membrane.^357^

##### Other ABCA transporters

The cystic fibrosis transmembrane conductance regulator (CFTR; ABCC7) correctors C13,^455^ C14,^455^ C17,^455^ genistein,^456^ and ivacaftor (Figure 2)^456^ were demonstrated to rescue *ABCA3* mutants by increasing total ABCA3 mutant protein levels,^455^ promoting subcellular targeting of ABCA3 into vesicular bodies,^455^ and improving lipid transport function of ABCA3.^456^ Furthermore, the correctors lumacaftor (VX-809; Figure 2), C3, and C4, and C18 increased the presence of ABCA4 at the cell membrane in ABCA4-overexpressing HEK293 cells, indicating promotion of ABCA4 trafficking to the plasma membrane.^457,458^ Promotion of trafficking has already been demonstrated for other ABC transporters, such as ABCC1^23,24^ and ABCC7.^459^ Hence, this mechanism represents a new potential therapeutic option for ABCA transporter-related AD. As proposed for ABCC7,^460^ the authors suggested a direct binding of the correctors to the ABCA4 protein,^457^ which has not yet been proven.

In an *Abca12* pig model of Harlequin ichthyosis, acitretin (Figure 2) treatment resulted in a redistribution of ABCA12 in the skin compared to wild-type pigs, and thus, a higher survival rate.^430^

#### Destabilizers of ABCA transporters

##### Natural compounds

In contrast to compounds that promote trafficking of functional ABCA1 to the plasma membrane, other compounds that have the opposite effect have been named ‘destablizers’. So far, only agents targeting ABCA1 are known. The lactone antibiotic brefeldin A (Figure 2) interfered with ABCA1 cell-surface localization, recycling, and intracellular trafficking.^387,461-463^ These effects were at least in part dependent on the interaction with brefeldin 1-inhibited guanine nucleotide exchange protein (BIG1).^461^ This interference reduced the functional fraction of ABCA1 and, consequently, ABCA1-mediated cholesterol and phospholipid transport.^255^ Similar observations have been made for the polyether-antibiotics monensin, which reduced ABCA1 turnover and trapped it inside endo- and lysosomes. Subsequently, monensin reduced the functional presence of ABCA1 at the cell surface,^464^ lowered cholesterol efflux,^463^ and increased intracellular cholesterol content.^463,464^ The same was demonstrated for nigericin, another polyether-antibiotic, which increased intracellular cholesterol concentration,^463^ and inhibited ABCA1-mediated cholesterol efflux from RAW264.7 macrophages.^385^ Inhibition of intracellular organelle transport as suggested for brefeldin A^387,461-463^ and monensin^463,464^ likely applies to nigericin as well.^463,465^ In addition, the endoplasmic reticulum stress promotor, tunicamycin, also reduced ABCA1 protein levels.^360,466^ This ‘downregulation’ is most likely mediated though stress-induced impaired ABCA1 trafficking and/or increased ABCA1 degradation.^466^ However, in terms of selective targeting of ABCA1 in particular, or ABCA transporters in general, these agents are less suitable as *in vivo* agents and serve better as *in vitro* controls.

The palmitic acid derivative 2-bromopalmitate (Figure 2) inhibited trafficking of ABCA1 to the plasma membrane and reduced ABCA1-mediated cholesterol efflux.^273,467^ However, the observed effect that ABCA1 did not translocate to the cell membrane in HEK293/*ABCA1* cells^467^ has not been demonstrated in BHK-21/*ABCA1* cells.^273^

##### Pharmacological drugs

Interestingly, the experimental anticancer drug serdemetan (JNJ-26854165) was demonstrated to induce *Abca1* mRNA levels but reduce ABCA1-mediated cholesterol efflux.^468^ The *Abca1* mRNA induction was due to induction of *Lxra* and *Lxrb*. The *Abca1* mRNA increase was also reflected at the protein level, which increased within 48 hours of exposure to serdemetan before a sudden decrease occurred. The authors also showed that ABCA1 turnover and degradation were increased. Thus, serdemetan can be considered a destabilizer.

##### Synthetic compounds

Cycloheximide was frequently used to interrupt intracellular trafficking of vesicles, including ABCA1 containing endo- and lysosomes.^387,464,468^

As mentioned earlier, ABCA1 is stabilized by *N*-acetyl-Leu-Leu-norleucinal.^316,386^ This stabilization could be abrogated by the protein kinase C inhibitor Gö6976, which affected not only ABCA1 protein content, but also cholesterol and phospholipid transport.^386^

## PART II: PIPELINE DEVELOPMENT TO GAIN NOVEL DIAGNOSTICS AND THERAPEUTICS

### *In silico* methodologies to predict novel lead structures

Rational drug design is the innovative process of identifying pharmaceutically relevant drug candidates. It is based on the information obtained in association with the drug target, *e.g.*, ABC transporters. In the following section, we will discuss computational approaches for *in silico* operations that help to identify novel lead molecules for potential diagnostic and therapeutic application.

#### Structure-based drug design

The development of computational methodologies for structure-based drug design to understand the relationship between transporter sequence/structure and function depends on the availability of structural as well as biological information. Recent advances in experimental approaches for structure determination have facilitated high-quality depictions of the structures of a growing number of ABC transporters in different conformational states.^469^ These experimental approaches include in particular X-ray crystallography and cryo-electron microscopy (cryo-EM).

**Figure 4.**
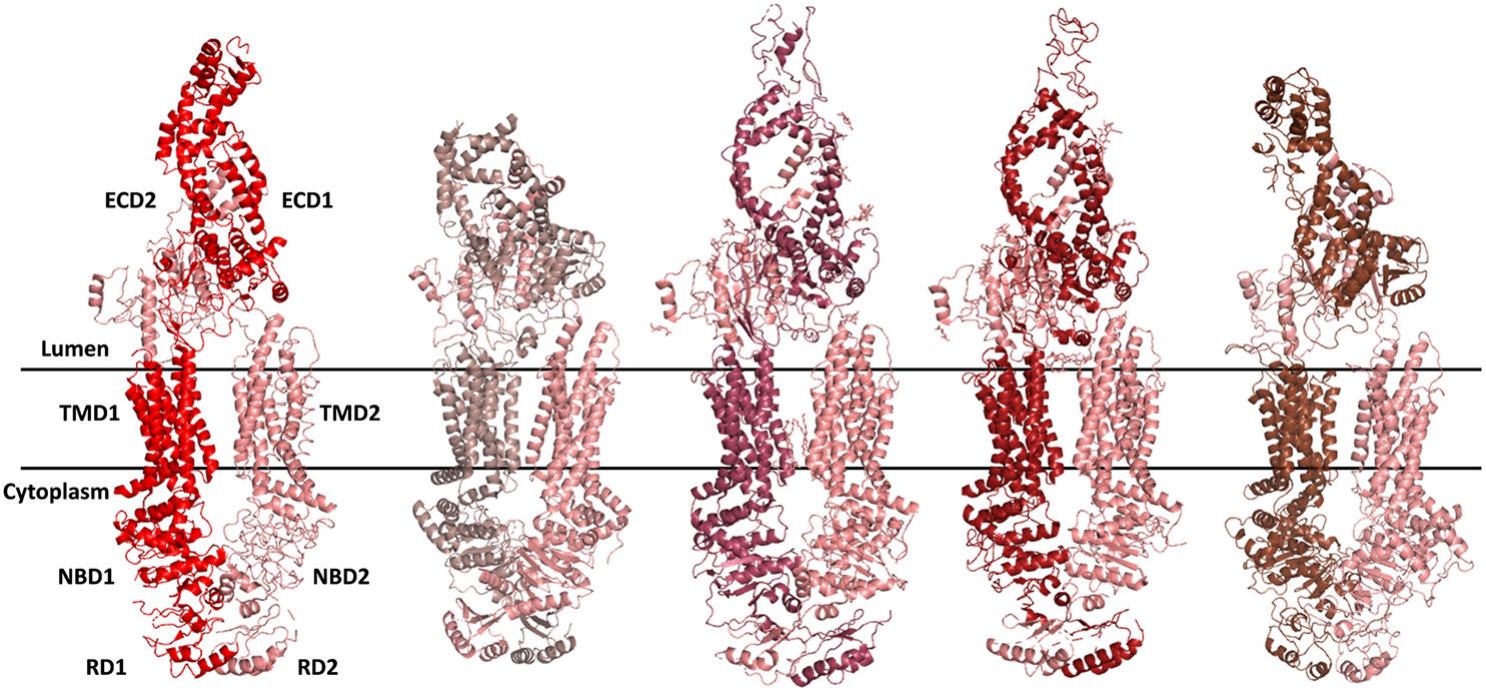
Available structures of ABCA transporters: the cryo-EM structures of human ABCA1^470^ (very left; PDB ID 5XJY) and ABCA4 [left (PDB ID 7LKP, middle (PDB ID 7E7I), and right (PDB ID 7M1Q)]^471-473^ as well as the homology model developed for human ABCA7 (very right).^475^ All three transporters are typical ABCA transporters with three crucial structural parts: two nucleotide-binding domains (NBDs; intracellular), two membrane-spanning domains [MSDs (2 x 6 transmembrane helices TMs); inter-membrane space], and two large extracellular domains (ECDs; extracellular).

Recently, the cryo-EM structures of human ABCA1^470^ and human ABCA4^471-473^ with resolutions of 4.1 Å and 3.3–3.6 Å, respectively, were reported. In addition, a cryo-EM structure of human ABCA7 has been announced^474^ on bioRxiv (biorxiv.org), which was, however, not published to this date (PDB ID: 7KQC). Nevertheless, a homology model of ABCA7 has been recently developed.^475^ Figure 4 shows the structures of ABCA1, ABCA4, and ABCA7 as determined by cryo-EM as well as homology modelling.

Considering the available structural knowledge, a ‘common’ ABCA transporter possesses a very long amino acid sequence (>2000 amino acids) and consists of two membrane-spanning domains (MSD1 and MSD2) each composed of six transmembrane helices (TM1–6 and TM7–12). These MSDs are followed by a cytoplasmic region comprising a nucleotide-binding domain (NBD1 and NBD2) and a small regulatory (R1 and R2) domain, which have been proposed to stabilize the interaction between NBD1 and NBD2^470,473^ and were found to strongly interact with each another in the absence of ATP.^471,472^

ABCA transporters are ‘type II transporters’ in which the MSDs indeed form a tunnel for substrate translocation from the cytosol to the lumen, however, represent separate entities without swapping/twisting of the MSDs, as this is the case with classical ‘type I transporters’ like ABCB1.^476^ Most TMs are completely exposed to the hydrophobic environment of the membrane, which could promote the attraction and binding of fat-soluble cholesterol as well as phospholipids before guidance to and through the substrate translocation tunnel, and which hosts several cholesterol and phospholipid binding sites.^470-474^

A unique feature amongst ABCA transporters in comparison to other ABC transporters is the existence of two large extracellular domains (ECD1 and ECD2). These domains together form a channel embedded in hydrophobic amino acids^470-472^ and are believed to facilitate intermediate storage of cholesterol^470^ and phospholipids. They have also been suggested as the primary binding site of APOA1,^471,477^ as indicated by the latest data on ABCA4.^471^ A large gap exists between the ECDs and MSDs, pointing to strong conformational changes that are required for ABCA transporter function.^470^ Another common feature amongst ABCA transporters are four intracellular and extracellular helices (IH1–4 and EH1–4), which are believed to provide the necessary flexibility for interaction between the MSDs and NBDs in the substrate translocation process,^478^ and were suggested to enable proper folding and function of these transporters.^471^

Of important note is that ABCA1 and ABCA4 share sequential and structural similarities with the ABCG family, in particular with ABCG5/ABCG8,^470^ which is the model type II transporter.^478^ This similarity suggests an evolutionary relevance amongst various ABC transporter subfamilies. More importantly, conserved sequential and structural similarities also support the translation of knowledge gained on other ABC transporter subfamilies to ABCA transporters.^470,472^ This is of particular interest when novel lead structures for new pharmacological targets, in this case under-studied ABC transporters,^18^ are focused,^6,18^ and specific binding sites located within the MSDs or NBDs are targeted.

Based on the sequence information of ABC transporters within the same family, homology-modeling techniques are the preferred choice for structure determination and binding site elucidation if these subtypes do not yield X-ray or cryo-EM structures. This methodology is of particular relevance for closely related homologs with high medical relevance,^198^ such as ABCA7 (similarity A1/A7: 54%; similarity A4/A7: 49%).^200^ The generated homology models can be refined further by molecular dynamics simulation, in which the transporter movement (‘trajectory’) is simulated to potentially unravel relevant transporter conformations. Very recently, potential ABCA1 drug binding sites have been proposed by this methodology,^479^ and an ABCA7 homology model has been developed for molecular docking experiments.^475^

Molecular docking is a very popular method for predicting binding orientations or poses of small-molecules within the transporter. Most often, the docking programs account for full conformational flexibility of ligands within the binding site, treating the protein as a rigid body. Binding site identification is an important prerequisite in the structure-based drug design implementation. In terms of ABC transporters, the search for binding hot spots and cavities on the entire volume of the protein (*e.g.*, through blind docking) is necessary due to the general lack of information on binding sites of ABC transporters.

Recently, in search of highly effective modulators addressing ABCG2-mediated MDR, derivatives of quinazolines were synthesized and biologically assessed using a Hoechst 33342 accumulation assay.^480^ By utilizing the cryo-EM structure of ABCG2,^481^ molecular docking studies were performed using a fragment-based approach.^482^ This approach was used to gain insights into the molecular determinants involved in the formation of the transporter-substrate complex.^480^ Based on the docking studies, the putative binding site of the ABCG2 substrate, Hoechst 33342, and its interaction with the amino acids in the binding pocket was proposed.^480^ The predicted binding pose was rationalized based on the mutagenesis data reported in the literature^483-487^ and further confirmed with kinetic studies to determine the mode of inhibition.^480^ This subsequent structure-based approach led to the discovery of highly potent pyrimidine-based ABCG2 inhibitors,^488,489^ specifically by identifying a novel binding pocket of this transporter.^488^ In terms of ABCA transporters, molecular docking experiments with the newly derived ABCA7 homology model applying a set of diverse pan-ABC transporter inhibitors revealed a putative common ‘multitarget binding site’ identified within the transmembrane domains of ABCA7. It must be noted that the nucleotide binding domains are the most highly conserved regions amongst all ABC transporters, and hence, may also represent a(nother) multitarget binding site for certain drugs. However, the vast majority of data reported in the past hint to the transmembrane domains as the actual venue of bioactivity in terms of ABC transporter modulation.^472^

These results as described above^475,480,488,489^ give this methodology a high relevance in the drug development process in terms of novel lead molecules in general, and provide the basis for rationally designed structure-guided approaches for the identification of modulators of ABCA transporters in particular, as recently demonstrated for ABCA7.^475^

#### Ligand-based drug design

##### Similarity search

The analysis of structure-activity relationships using ligand-based approaches is an essential component of medicinal chemistry and pharmacology of ABC transporters. This becomes evident as X-ray or cryo-EM structures of most ABC transporter subtypes are lacking to serve as suitable templates with sufficient similarity for generating homology models. Ligand-based approaches establish a correlation between the molecular structure of a small-molecule and the triggered biological response of the target. The chemical representation of the molecules is often expressed using descriptors, which are attributes that conserve the physicochemical information of the molecule. These descriptors refer to generic properties such as LogP, molecular weight, polar surface area, rotatable bonds, or molar refractivity. Alternatively, structural representations of the molecules can form fingerprints that portray existent molecular features of the molecule in a binary code. These fingerprints are, for example, path-like,^490^ or circular-based,^491,492^ such as MACCS or ECFP4, respectively. Utilizing these representations of molecules, similarity-driven virtual screenings can be applied. Here, molecules are extracted from a virtual library of millions or billions of compounds compared to the bioactive template molecule(s) according to the similarity principle. The abstract representation of molecules enables clustering of compounds, which is a methodology to categorize a diverse set of molecules. Moreover, these abstract representations can be used in different machine learning (artificial intelligence) approaches.

##### Pharmacophore modelling

Another common approach is pharmacophore modelling, which analyzes a number of ligands with a common mechanism of action. The model is the ensemble of common chemical features that are required to ensure the molecular interaction of the ligands with the target, such as hydrogen bond donors and acceptors as well as aromatic and hydrophobic centers. The pharmacophore models are generated by extracting common molecular features through flexible alignment of the active biomolecules.^493,494^ This can be achieved by generating all possible conformations of the ligand and aligning them to determine the essential chemical features and molecular orientation to construct the pharmacophore model. The conformational flexibility of the ligands representing the chemical features is the key factor in the pharmacophore model generation.

##### Pattern analysis

In addition to these classical computational approaches, similarity search and pharmacophore modelling, a pattern analysis approach (‘C@PA’ = computer-aided pattern analysis’) has been reported recently.^18,19,495^ Pattern analysis extracts both basic scaffolds and the statistical distribution of substructural elements amongst the template ligands. It works similarly to non-physicochemical properties-related fingerprints and conserves substructural features as they are present in the molecules. Pattern analysis has specifically been derived for the development of novel potent multitarget ABC transporter inhibitors. The basic operations were the categorization of bioactive molecules according to their inhibitory power against specific ABC transporters and their classification according to their selectivity profile. The respective classes can statistically be analyzed for both their basic scaffolds and/or their substructural composition to extract the desired pharmacological profile and target preferences. The generated model focused multitargeting of ABC transporters, and resulted in a biological hit rate of 21.7%.^19^ Adaption of the model (‘C@PA_1.2’) through additional non-statistical and exploratory measures increased the biological hit rate to 40%,^18^ and an additional extension of the model enabled the discovery of the ‘outer multitarget modulator landscape’, which represented weak multitarget bioactivities (>10 μM) supporting the discovery of a larger number of multitarget agents.^495^ The hit rates are impressive considering that this approach takes several targets with individual ‘ligand preferences’ into account. Furthermore, as several ABC transporters of distinct subfamilies were considered (ABCB1, ABCC1, ABCG2), the resultant multitarget agents open up the possibility to explore under-studied ABC transporters,^18^ in particular ABCA transporters in terms of AD.^6,14^

##### Combined approaches

Apart from the individual use of these methodologies, combined approaches may lead to improved hit rates and better prediction capabilities with respect to bioactivity of small-molecules. This has in particular been demonstrated for a combined virtual screening approach using similarity search and pharmacophore modelling for the discovery of novel ABCC1 inhibitors.^493^ Also, certain pattern analysis approaches have used a data set derived from a similarity search and pharmacophore modelling approach, and hence, can also be considered a combined computational approach.^18,495^

### *In vitro* methodologies to assess novel lead structures

The previous sections have already outlined the diverse testing systems that have been used to assess the modulatory effects of effectors toward ABCA transporters. The following section will highlight the ABCA transporter-expressing host systems and the related assays that can be implemented into the pipeline for the assessment of novel lead molecules as potential ABCA transporter diagnostics or therapeutics.

#### Host system of ABCA transporters

The transporter host system (ABCA transporter carrying unit) can be categorized into (i) living-cell-based or (ii) membrane preparation-/vesicle-based (including isolated and reconstituted proteins). The vast majority of biological investigations used living cells. Here, two different living cell-based transporter host systems can be differentiated: (i) native/induced/selected cells and (ii) transfected cells.

##### Native ABCA transporters-expressing living cells

Native/induced/selected cells naturally express the respective ABCA transporter or have been exposed to a ‘standard’ inducer, for example, the ABCA1 inducers 22-(*R*)-hydroxy-cholesterol,^122,205,249,252,259,262-264,268,277,278,305-315^ TO901317,^205,245,250,252,259,260,262,264,271,272,279,280,282,308,310,317,319,322,324,326,328-345^ or 8-Br-cAMP,^230,249,255,266,290,292^ and overexpress the respective transporter in response (*e.g.*, ABCA1). Most commonly, human or murine cells have been used. Table 4 summarizes the cell lines used to assess the ABCA transporter modulators discussed in the previous sections. It must be noted that the addressed pathways regulate also the overexpression of other ABC transporters. In terms of the studies of ABCA1, the co-expression (*i.e.*, co-upregulation and co-downregulation) of other members, such as ABCG1, has frequently been observed.^160,320,335,364,366,402,410,418,421,448^

In terms of ABCA1, most studies have been conducted with human THP1,^231,245,249,256,268,272,275,292,308,310,312-316,321,328,335,338,339,341,342,360,363,364,366,377,384,388-397^ murine J774.A1,^252,254,255,259,265,271,278,289-292,384,392,393^ or murine RAW264.7 macrophages.^230,249,312,313,321,336,339,342,352,360,365,367,369,375,376,381,385,399,402,404,406,408,410,416-419,421,424,425,438,442,448^ In the set-up of a drug development pipeline, these cell lines are the backbone of the *in vitro* assessment of potential candidates.

Regarding other ABCA transporters, the situation is much more complicated due to the lack of cell lines that naturally (and almost exclusively) express the respective ABCA transporter. Consequently, these ABCA transporters are much less studied and well-established. However, transfected cell lines are of great help to study one particular transporter instead of using native cell lines that may co-express several members.

##### ABCA transporters-transfected living cells

In terms of ABCA1, cell lines transfected with human *ABCA1* have often been used, *e.g.*, human embryonic kidney (HEK) cells (HEK293/ABCA1)^171,201,202,249,260,267,270,275,329,352,386,464,467,498,499^ and baby hamster kidney (BHK) cells (BHK-21/ABCA1).^230,245,273,292,422^ These transporter host systems have also been used to study other transporters, ABCA2,^498,500^ ABCA3,^235,241,498^ ABCA4,^133-136,201,457,458,501,502^ ABCA5,^503^ ABCA7,^201,202,386,422,498^ ABCA8,^10^ ABCA12,^498^ and ABCA13.^48^

Transfected cells often express lower levels of the introduced transporter than native cell lines, which is a problem if the host cell lines (*e.g.*, HEK or BHK-21) naturally express other ABC transporters as well. However, these transporter host systems are suitable to confirm results, and might be the only possibility to address ABCA transporters other than ABCA1.

##### Isolated ABCA transport proteins

Finally, apart from intact cells, vesicles of enriched or purified/reconstituted ABCA transporters have also been used to assess transporter function. Compared with living-cell based assays, this kind of host system is rarely represented in the literature regarding ABCA transporters.^133-139,201,499-502,504-506^ Specifically ATPase assays are popular to assess functional ABC transporter modulation.^23,24,507-510^ While transport protein purification and reconstitution in vesicles or nano discs requires advanced engineering, and is expensive and resource-consuming, membrane preparations of transporters, in particular for ATPase assays, are much more feasible. However, this method has been used somewhat scarcely for ABCA transporter function assessment.^133-135,137-139,201,499-502,504-506^

#### Functional assessment of ABCA transporters

Two groups of tracers have been established in terms of ABCA transporter function: (i) radiolabeled substrates,^250,272,305,306,338,339,354,364,366,393,395,404,419,511,512,136,222,230,245,249,253,255,259,260,262,264,265,267-270,273,276,278,289-292,311,313,315,318,329,341,367,377,381,384,385,464,467,499,513^ and (ii) fluorescent substrates.^171,201,238,251,252,254,256,258,261,271,282,308,319,321,330,332,335,342,360,379,389,390,392,397,402,406,455,456,468,514-518^

##### Radiolabeled tracers of ABCA transport function

In terms of radiolabeled substrates, cholesterol is by far the most frequently used genuine ABCA1 substrate,^230,245,249,255,260,264,265,267-270,272,273,276,278,289-292,305,306,313,315,318,329,338,339,354,366,367,381,384,385,393-395,404,408,419,464,467,499,512^ followed by phospholipid(-components).^249,255,267,269,273,311,464,467,514^ However, other substrates have also been used. These substrates include mostly molecules with sterane scaffold, such as β-sitosterol (ABCA1)^262^ and estradiol-β-glucuronide (ABCA8).^222^ Moreover, lipid-like substrates have attracted attention, like sphingosine-1-phosphate (ABCA1),^229,496^ α-tocopherol (ABCA1),^230^ and ATRA (ABCA4).^136^ Notably, radiolabeled substrates are very effective in terms of accurate tracing of protein function, as these molecules are not changed in their molecular integrity in contrast to fluorescence probes.^171,201,238,251,252,254,256,258,261,271,282,308,319,321,330,332,335,342,360,379,389,390,392,397,402,406,455,456,468,514-518^ On the downside, conducting these experiments is constrained to regulatory requirements and requires extensive staff training as well as expensive safety measures and laboratory equipment.

##### Fluorescent tracers of ABCA transport function

Regarding fluorescent derivatives of cholesterol and phospholipids, two major types can be differentiated: (i) 7-nitro-2,1,3-benzooxadiazole (NBD) derivatives^201,251,252,254,256,258,261,308,335,342,360,379,389,390,392,394,397,402,406,408,468^ and (ii) 4,4-difluoro-4-bora-3a,4a-diaza-*s*-indacene (BODIPY) derivatives.^271,282,319,321,330,332,455,456,515-517^ Other fluorophore-labeled dyes have been reported, too, including the sterane analog dansylestramustine,^171,238,518^ and propargyl choline, which is processed *in vitro* into propargylated phospholipids.^514^

In addition to the stated fluorescent tracers of ABCA transport function, several other derivatives of other substrates can be proposed. For example, *N*-3-oxododecanoyl-*L*-homoserine lactone (3OC12-HSL) was suggested as ABCA1 substrate, but final proof was missing.^519^ Thus, it may be a suitable candidate for validation in a new set-up *in vitro* assay for ABCA1 (and potentially other ABCA transporters). Other examples of potential probes are fluorescenct dyes that stand in association with cellular cholesterol and phospholipid distribution and ABCA1-mediated cholesterol and phospholipid transport.^516^ These include, for example, β-BODIPY FL C5-HPC, β-BODIPY FL C12-HPC, BODIPY TR ceramide, and Red/Green BODIPY PC-A2, amongst many others.^520-522^

Fluorescenct dyes are well-established tracers of ABC transporter function,^18,19,23,24,284,480,488,489,493,507,523-525^ and the knowledge that has accumulated regarding the well-studied ABC transporters ABCB1, ABCC1, and ABCG2 can be transferred to ABCA transporters as well. However, the added fluorophore changes the molecular composition of the tracing molecules. This alteration inheres the potential risk of changing affinities and even the binding site(s) of these molecules, undermining functional-kinetic analyses regarding binding site determination and elucidation of the mode of action. Nevertheless, fluorescence probes are – if used and established correctly – extremely reliable, and can be used without regulatory restrictions and necessity of special equipment, except for microplate readers and/or flow cytometers.

##### Colorimetric determination of ABCA transport function – ATPase assays

As mentioned above, ATPase assays have also been used to functionally analyze ABCA transporter function, in particular for ABCA1,^201,499,505,506^ ABCA2,^500^ ABCA3^139,504^ ABCA4,^133-135,137,138,201,501,502^ and ABCA7,^201^ although this methodology has been used somewhat rarely compared to other functional approaches. ATPase assays are based on the principle that the active transport of any substrate of ABC transporters consumes energy. This energy is derived from the cleavage of ATP to ADP and P_i_, and can be detected by different methodologies.^23,24,507-510,526^ Table 5 highlights known ATPase modulators of ABCA transporters and the associated literature reports.

ATPase assays have been and still are popular in terms of functional ABC transporter modulation in general.^23,24,507-510^ Strikingly, the NBDs of ABC transporters are – in contrast to the various binding sites identified within the transmembrane domains of ABC transporters^475^ – highly conserved. This conservation enables targeting of ABCA NBDs by known ATPase modulators of other ABC transporters. Therefore, ABCA transporter function can be detected by methodologies that have already been established for other ABC transporters.^23,24,507-510,526^ This transfer of knowledge will be of great use to confirm obtained results from other functional ABCA transporter analyses.

##### Colorimetric determination of ABCA transport function – other detection methodologies

As a final note, it must be mentioned that other colorimetric analyses were also used to quantify the ABCA transporter-mediated function, specifically for transport of cholesterol or choline-containing lipids, using commercially available assay kits.^202,205,246,251,268,272,275,329-332,334,336,354,365,366,369,372,374-376,386-389,392,405,406,416,418,421,422,424,425,441,511^ However these methodologies require time-consuming extraction processes of the lipids, and hence, are less suitable to track the function of ABCA transporters in real-time and to determine kinetic aspects of their cholesterol and lipid transport.

In rare instances, the extraction of lipid components was accomplished after incubation with a radioactive marker.^246^ While this is a valid methodology to accurately determine lipid components within cells, it increases workload and attracts regulatory constraints.

Gas-liquid chromatography has also been used in some reports.^353,355^ An extraction-free staining of cholesterol inside of cells was also demonstrated (filipin III^251,331,333,341,358^ or Oil Red O staining ^330,332,364,366,369,375,388,389,392,393,399,402,410,417,421,425,448^

However, these systems are not suitable to track single-cell ABCA-mediated cholesterol or phospholipid transport.

##### Quantification of ABCA transporter regulation

Besides qPCR and western blotting, ABCA transporter expression was reported in several studies using fluorimetric assays. This was accomplished with either (i) green fluorescent protein-(GFP)- tagged/labelled ABCA transporters^235,241,261,275,386,422,464,504^ or (ii) luciferase promotor-(LUC)-transfected^271,309,319,352,367,379,381,405,406,419,436,447^ ABCA transporter cells in luciferase reporter gene assays.

### *In vivo* assessment of clinical candidates

*In vivo* models play a key role in drug discovery. Although *in vitro* and cellular models are less expensive and less time consuming, *in vivo* models are needed to test ABCA modulators under physiological conditions. Safety, toxicity, and efficacy of a drug candidate must be tested in an *in vivo* model as a last step before transferring it to clinical evaluation. However, these models also have disadvantages. Animal studies are time consuming and require advanced personnel training and resources for maintaining the animals. In addition although they are closer to humans than *in vitro* models, there are considerable physiological differences between species with respect to drug absorption, metabolism, and excretion, which may impede translatability. Furthermore, the use of animals in research has its ethical concerns. Thus, in recent years, research has been directed to reduce animal use and increase animal welfare.

*In vivo* models have previously been used to study the role of ABCA transporters in physiology and disease as described above. Thus, there are already available animal models for testing of ABCA modulators for the most prominent subtypes (Table 6). As stated above, these models represent the last step before clinical evaluation of potential small-molecule therapeutics in humans. Thus, after *in silico* identification and *in vitro* assessment, these *in vivo* models are the third column in the development of novel ABCA transporter diagnostics and therapeutics. In the following section, different *in vivo* models will be described in more detail.

#### Knock-out mouse models

A genetic knock-out mouse model is an animal model in which one or more genes of interest have been deactivated or removed by means of gene targeting. Knock-out animals allow for direct investigation of the effect of a specific gene in an organism, as the loss of gene activity often causes phenotypic changes uncovering the function and biological mechanism of the targeted gene.^535^ Knock-out mice have become one of the most useful scientific tools to analyze the human genome and its potential roles in many diseases.^535^ Thus, knock-out animals are currently essential experimental tools for the investigation of genetic disorders and the evaluation of novel drugs.^536^ Furthermore, the current knowledge on genome editing using the CRISPR/Cas9 system makes generation of knock-out lines considerably faster than with the use of embryonic stem cells. To no surprise, this method has quickly become the most powerful tool for generating genetic models.^537^

Knock-out animal models are designed with two variables in mind: (i) where and (ii) when is the gene of interest deactivated. The simplest and most common approach is a constitutive, ubiquitous knock-out, *i.e.*, the product protein is absent permanently in all cells of an organism. To overcome limitations of this broad approach, more refined models have been developed. These conditional models use Cre-Lox recombination to target a gene either in specific cell populations, at specific time points, or a combination of both. Here, the target gene is modified by inserting two loxP sites. The flanked gene segment can then be excised by the Cre recombinase. Cre activity, *i.e.*, gene knock-out, can be limited to certain cell populations by appropriate promotor choice and/or linked to a tamoxifen-responsive element to control the exact time point at which the knock-out is induced.

Until now, several *Abca* animal knock-out models have been described, which are summarized in Table 6. These models are mainly mouse lines, except for *ABCA13* (monkey).^534^ These animal models have contributed fundamentally to identifying the role of ABCA transporters in physiological conditions as well as in disease pathogenesis. In addition, these models can be used for novel drug testing, as they provide information about target specificity. If a drug is 100% specific for a transporter, knock-out of this transporter should completely abolish the drug’s effects observed in naïve animals. However, gene knock-outs often have phenotypical effects *per se* that need to be taken into account when evaluating drug effects.

#### RNAi models

The use of RNA interference (RNAi) is an alternative to knock-out models. This technique is based on post-transcriptional silencing of the targeted gene using siRNA molecules that are designed to bind to the target mRNA.^538^ This process will deactivate the mRNA using the cell’s own defense mechanism against pathogens. In contrast to standard knock-out models, this silencing is temporary as the siRNA molecule will be degraded but the gene transcription continues.^527^

To avoid this temporal limitation, short-hairpin RNA (shRNA) has been developed. This method is based on the use of vectors that incorporate into the cell DNA and encode for shRNA. After transcription, these vectors are processed into siRNA. These shRNAs are continuously transcribed, increasing reproducibility of results.^539^

#### Overexpression models

Similar to knock-out models, overexpression models can be used to investigate the function of a gene by evaluating the resultant phenotype. In addition, overexpression models have long been used for modeling diseases such as AD^540^ or PD.^541^

In the investigation of ABCA transporters, these models can resemble the effect of chronic activation of the transporters and may help to identify its physiological functions by evaluating the pathways upregulated in comparison to control animals.^127^

#### Humanized ABC transporter mouse models

Before it can be translated into clinical practice, each novel drug candidate must be tested in an *in vivo* model. However, the translational value of the animal model largely depends on whether the disease pathway under investigation is conserved between the two species. Therefore, replacing the original (*e.g.*, murine) gene by the respective human gene likely improves the animal model, and thus, is beneficial for evaluating a novel drug’s efficacy and specificity in clinical practice.^542^ With this approach, mice can be used as tools for pre-clinical screening and efficacy evaluation of new drugs, given their improved ability to predict human responses to treatments.

Our group has previously established a humanized *ABCC1* mouse model,^543^ and an *ABCA7* model is under characterization. Here, we generated knock-in mouse models producing a chimeric protein that is completely human except for one amino acid.^543^ In addition, as this gene was flanked by loxP sites, this humanized model can be knocked out in specific cell populations and at a specific age.^543^ Models such as these represent the future of pre-clinical drug candidate evaluation.

In addition, Dallas *et al.* successfully generated a humanized *ABCG2* mouse model.^544^ However, other models, such as humanized *ABCB1* mice, were not successful despite multiple attempts.^545^

#### Disease models

In addition, all the models described above can also be used to study the role of a gene for the pathophysiology of specific diseases. For example, *Abca* knock-out models have been crossed with transgenic mice in order to study their potential role in AD.^54,123,131,161-163,527^ These studies have elucidated potential disease mechanisms involving ABCA transporters that cannot be studied in patients.

Moreover, once a drug is developed and its specificity is proven, disease models enable evaluation of the role of that specific transporter in the pathophysiology of the disease. At the same time, these results may be the first step to evaluate the potential of novel transporter modulators as therapy for the respective disease.

#### Imaging techniques

Lastly, *in vivo* imaging can be used for the development of new drugs. On the one hand, labeling drug candidates with radioactive isotopes can give information about the drug distribution, drug target, and drug metabolism *in vivo*. In addition, it can also show whether a drug is able to cross specific natural barriers, such as the BBB. *In vivo* imaging can help to select candidates that appear successful or to discard drugs that seem likely to fail.^546^

On the other hand, drug candidates can also be used to develop new radiotracers (*e.g.*, PET tracers) targeting ABCA transporters that could then be used in clinical diagnostics. Radiotracers would facilitate the study of the specific gene and/or its product protein in human patients *in vivo* and in a longitudinal fashion, enabling a much better understanding of the role of ABCA transporters in human (patho)physiology.^547^ In this regard, knock-out animals can be used as negative controls for the development of new ABCA radiotracers to evaluate the specificity of the radiotracer.^548^ Furthermore, these very same radiotracers can be used in animal disease models, enabling longitudinal studies and reducing the number of animals required.^549-551^

## CONCLUDING REMARKS: WHERE DO WE GO FROM HERE?

Several *in vivo* studies demonstrated that modulators of ABCA transporters, in particular ABCA1, have systemic effects.^231,249,250,253,271,275,289,293,297,330,335,344,350,361,362,366,368-370,376,378,383,410,415,417-419,425,431,436,448^ However, the vast majority of these modulators were regulators,^231,250,253,271,297,330,335,344,350,361,362,366,368-370,376,378,383,410,415,417-419,425,431,436,448^ specifically inducers,^250,253,271,297,330,344,361,362,366,368-370,376,378,383,410,415,418,419,425,431^ and only very few interactors demonstrated *in vivo* effects.^249,289,293^ Mostly emphasizing atherosclerosis,^249,275,289,366,369,370,378,410,417-419,425,431,448^ these regulators were able to demonstrate that cellular and plasma lipid content^249,271,275,289,330,366,369,378,419,425,431^ as well as atherosclerotic plaque formation^275,289,366,369,370,410,417-419,425,448^ could be changed compared to controls (enhanced or reduced) after treatment with the respective drug. Only very few *in vivo* approaches targeted for AD.^293,297,344,383^

Taking the challenge of CNS penetration of these drugs into account, drugs active in atherosclerosis models could generally be suggested to also have certain therapeutic relevance regarding AD. Nevertheless, so far, none of these drugs has made it into clinical evaluation in humans. The underlying cause can be pinned to the fact that the principal mechanism by which ABCA transporters contribute to AD is still unknown. While a rationale can be found in atherosclerosis (efflux of cellular lipid to APOE and HDL resulting in lower lipid burden in the vascular system), the translation of this rationale to AD can only be achieved to a very limited extent. Several questions need addressing in future evaluations: (i) what is the general function of ABCA transporters in the brain to ameliorate (or exacerbate) AD in patients; (ii) when does this development start; and (iii) at which stage of development can a pharmacological intervention with ABCA transporter modulators lead to a positive therapeutic effect?

In this regard, more *in vitro* tests are needed with new lead structures that are rigorously assessed for their particular mechanism of action – to study *vice versa* the mechanism of action of ABCA transporters in general. One possibility to gain novel lead structures is the screening of huge analog compound libraries. However, the number of existing compounds is limited, and blind *in vitro* testing is resource-consuming, especially regarding time and funds. Computational methodologies may help to generate novel lead structures based on the knowledge of existing modulators of ABCA transporters. This has led to new lead molecules in the past.^18,19,493,495^ Particularly the knowledge on ABCA1 and ABCA8 inhibitors and substrates is of interest, because these compounds inherit the molecular-structural information that is critical for direct binding to these transporters. Considering the newly developed pattern analysis methodology, C@PA,^18,19,495^ the scaffolds and substructural composition of this set of molecules may reveal the critical necessities for direct interaction with ABCA transporters. C@PA is therefore of high relevance because it was specifically developed to gain multitargeting pan-ABC transporter modulators^18,19,495^ – molecules that particularly interact with different ABC transporters of different subfamilies. Assuming that a conserved multitarget binding site exists as proposed earlier,^6,14,475^ multitargeting may be the key to explore under-studied ABC transporters in general and ABCA transporters in particular.^6,14,18,19^ Several thousands of these molecules have already been predicted,^18,19,493,495^ and the predictions were in part biologically confirmed.^18,19,493,495^ Additionally, selected pan-ABC transporter inhibitors were analyzed in molecular docking studies, which revealed the potential existence of the multitarget binding site.^475^ Hence, combining the existent knowledge of ABCA transporter modulators with (sub)structural elements of these pan-ABC transporter modulators and powerful computational approaches (*e.g.*, molecular docking or molecular dynamics simulations) could ultimately lead to the successful exploration of ABCA transporters in general, as well as ABCA1 and ABCA7 in particular.^28,95,103-112^

Several drugs and drug-like compounds have already been demonstrated to be pan-ABC transporter modulators interacting also with ABCA transporters. These drugs and drug-like compounds are, for example, cyclosporine A (9 targets of 4 subfamilies: ABCA1,^245^ ABCB1,^20^ ABCB4,^552^ ABCB11,^553^ ABCC1–2,^24,554^ ABCC10,^26^ and ABCG1–2^555,556^), glibenclamide (8 targets of 4 subfamilies: ABCA1,^270^ ABCB11,^553^ ABCC1,^24^ ABCC5,^557^ ABCC7–9,^558-560^ and ABCG2^554^), imatinib (6 targets of 4 subfamilies: ABCA3,^426^ ABCB1,^561^ ABCB11,^553^ ABCC1,^561^ ABCC10,^561^ and ABCG2^561^), probenecid (8 targets of 2 subfamilies: ABCA8,^222^ ABCC1–6,^24,26,562-564^ ABCC10^565^), verapamil (9 targets of 4 subfamilies: ABCA8,^222^ ABCB1,^20^ ABCB4–5,^552,566^ ABCB11,^567^ ABCC1,^24^ ABCC4,^568^ ABCC10,^565^ and ABCG2^554^), and verlukast (11 targets of 4 subfamilies: ABCA8,^222^ ABCB4,^552^ ABCB11,^553^ ABCC1–5,^24,554,557,564,569^ ABCC10–11,^26,570^ ABCG2^554^). *In silico* analyses with verapamil and verlukast supported the notion of addressing the multitarget binding site in ABCA7.^475^ Taking their structural peculiarities in a pattern-based rational drug design approach into account may yield novel lead structures for functional *in vitro* studies of ABCA transporters. This may ultimately result in the development of innovative AD diagnostics and therapeutics.

## Figures and Tables

**Table 1.** ABC transporters and related neurological and psychiatric diseases.

ABC transporter	Associated diseases
ABCA1	AD^50^ HD^51^
ABCA2	AD^52^ abnormal sphingolipid metabolism^53,54^
ABCA4	cone-rod dystrophy^55^ fundus flavimaculatus^56^ retinitis pigmentosa^57,58^ Stargardt disease^59-62^
ABCA5	AD^28^
ABCA7	AD^63^
ABCA13	Lewy body disease^64^ psychiatric disorders^48,65,66^ stroke *in mice*^67^
ABCB1	AD^28^ brain tumors^68^ HIV-associated depression and schizophrenia^69,70^ HIV-associated encephalopathy^46^ epilepsy^71^ ischemic stroke^72^ MS^35^ multiple systems atrophy^73^ PD^74^ progressive supranuclear palsy^75^ Creutzfeldt-Jakob disease^76^
ABCB7	PD^77^
ABCB9	PD^78^
ABCC1	AD^28^ brain tumors^79^ epilepsy^39^ HIV-associated encephalopathy^45^ ischemic stroke^80^
ABCC2	brain tumors^79^ epilepsy^39^
ABCC3	brain tumors^79^ epilepsy^39^
ABCC8	ALS^81^
ABCC9	ALS^81^ limbic-predominant age-related TDP-43 encephalopathy (LATE)^82^ hippocampal sclerosis of aging and depression^83^
ABCD1	cerebral adrenoleukodystrophy^84^
ABCG1	AD^85^ brain metabolic disorder^86^
ABCG2	AD^87^ ALS^88^ brain tumors^89^ epilepsy^90^ MS^91^ PD^47^ traumatic brain injury^92^
ABCG4	AD^93^ HD^51^

**Table 2.** Currently known modulators of ABCA1.

Mode of modulation	Name of modulator	Effect concentration; concentration range; EC_50_; dose; ED_50_
(Potential) substrates	cholesterol	-
	phospholipids	-
	β-sitosterol	-
	sphingomyelin	-
	α-tocopherol	-
Activators	ATI-5261	1.07 μM; 30 mg/kg body weight *in mice*
	CS-6253	0.73 μM; 20 mg/kg body weight *in mice*
Inhibitors	BLT-4	150 μM
	bromosulfophthaleine	500 μM
	bumetanide	200 μM
	cyclosporine A	1–20 μM; IC_50_ = 5.1–7.6 μM
	DIDS	40–500 μM
	diphenylamine 2-carboxylic acid	500 μM
	flufenamic acid	500 μM
	furosemide	200 μM
	glibenclamide	50–1000 μM
	pimecrolimus	20 μM; IC_50_ = 7.0 μM
	probucol	1.9–20 μM
	sirolimus	20 μM; IC_50_ = 18.8 μM
	tacrolimus	20 μM; IC_50_ = 13.6 μM
	valspodar	5 μM; IC_50_ = 1.9 μM
Inducers	A-769662	250 μM
	aclarubicin	EC_50_ = 0.49 μM
	allicin	2.5–10 μM
	cAMP	0.1–10 μM
	butyryl-cAMP	300 μM
	8-Br-cAMP	0.3–1000 μM
	CPT-cAMP	300–500 μM
	atorvastatin	5–10 μM; 4 mg/kg body weight *in mice*
	ATRA	0.25–10 μM
	AZ1–AZ9	ED_50_ = 1.49–341 μmol/kg body weight *in mice*
	AZ-1	10 μM
	AZ-2	10 μM
	AZ10606120	10 μM
	AZ876	ED_50_ = 0.956 μmol/kg body weight *in mice*
	BCD1	EC_50_ = 0.035 μM
	*N-benzothiazolyl-2-benzenesulfonamides*	EC_50_ = 0.37–33.42 μM
	berberine	5–20 μM
	bergapten	12.5–50.0 mg/kg body weight *in rats*
	bexarotene	0.1–1 μM
	bezafibrate	10–200 μM
	BMS-852927	ED_50_ = 2.10 μmol/kg body weight *in mice*
	sodium-butyrate	1000–10.000 μM; 200–400 mg/kg body weight *in mice*
	cholesterol	12.9–100 μM
	cholic acid analog 14b	5–40 μM
	celastrol	0.1–1.0 μM; 0.5–1 mg/kg body weight *in mice*
	*chalcone derivatives*	5–10 μM; 20 mg/kg body weight *in mice*
	*chromene derivatives 2, 3, and 5*	25 μM
	chromone analog 6	25 μM
	CL2-57	10 μM; 10 mg/kg body weight *in mice*
	curcumin	5–40 μM
	daidzein	EC_50_ = 3.17 μM
	danthron	10–40 μM; 60 mg/kg body weight *in mice*
	1,6–*O,O*-diacetylbritannilactone	8–10 μM; 10 mL/kg body weight *in mice*
	digoxin	0.010 μM
	doxazosin	10 μM
	doxorubicin	0.0316–1 μM; 20 mg/kg body weight *in mice*
	efatutazone	40 μM
	E3317	0.01–1 μM; EC_50_ = 0.2 μM
	EGCG	40 mg/kg body weight *in mice*
	homo-eriodictyol	41.4–165 μM
	ethyl 2,4,6-trihydroxybenzoate	50–100 μM
	F1	ED_50_ = <30 μmol/kg
	F4	10 μM
	fargesin	20 μM; 50 mg/kg body weight *in mice*
	fenofibrate	2.77–40 μM
	fluvastatin	1–20 μM
	FPD5	1 μM; 0.005–0.02 mg/kg body weight *in mice*
	fucosterol	100-200 μM
	geniposide	515 μM; 50–100 mg/kg body weight *in mice*
	ginsenoside (*derivatives*)	10–30 μM
	ginsenoside compound K	1.25 μM
	glycyrrhizine	60.8–243 μM
	GQ-11	20 mg/kg body weight *in mice*
	GW3965	0.5–50 μM; ED_50_ = 0.969 μmol/kg body weight *in mice*
	GW7845	5 μM
	*gypenosides*	5 μg/mL
	hesperetin-7-*O*-3-_d_-glucopyranoside	107–431 μM
	hesperetin-7-*O*-rutinosid	100 μM; 3 mg/kg body weight *in mice*
	20-(*S)*-hydroxycholesterol	5–20 μM
	4-hydroxycholesterol	1–20 μM
	22-(*R*)-hydroxycholesterol	1–25 μM; EC_50_ = 1.0 μM
	22-(*S*)-hydroxycholesterol	5–20 μM
	24-hydroxycholesterol	20 μM
	24-(*S*)-hydroxycholesterol	0.5–1.5 μM
	25-hydroxycholesterol	2–12.4 μM
	27-hydroxycholesterol	6.21 μM-10 μM
	3-hydroxytyrosol	2–5 μM
	idarubicin	0.1 μM
	kaempferol	2.5–10 μM
	L836,978	*u.c.* ^a^
	kuwanon G	20 μM
	L-839,867	0.1–1 μM
	LXR623	0.1–1 μM; ED_50_ = 31.5 μmol/kg body weight *in mice*
	lycopene	2.2–6.6 mg/kg body weight *in ferrets*
	M2	10 μM
	maslinic acid	20 μM
	metformin	10 μM
	mevalonate	5–500 μM
	mevastatin	50 μM
	mitotane	20–50 μM
	naringenin	25–100 μM
	obeticholic acid	40 mg/kg body weight *in mice*
	ondansetron	1 μM
	orlistat	50 μM
	ouabain	0.010 μM
	paeonol	100 μM
	PCB29-pQ	5–10 μM
	pemafibrate	0.1–10 μM; 0.3 mg/kg body weight *in mice*
	pestalotioquinoside C	50 μM
	phenethyl isothiocyanate	30–75 mg/kg body weight *in mice*
	*Tadehagi triquetrum*-derived *glycosides*	10 μM
	pioglitazone	5–10 μM; EC_50_ = 1.28–7.474 μM; 20 mg/kg body weight *in mice*
	pitavastatin	0.1–10 μM
	platycodin D	5–20 μM
	PMA	0.32 μM
	ponasterone A	2–5 μM
	pratensein	EC_50_ = 2.91 μM
	propofol	50 μM
	prostaglandin J2	1–20 μM
	pyrrole-imidazole-polyamide	1 μM; 1 mg/kg body weight *in mice*
	pyrromycin	EC_50_ = 0.85 μM
	quercetin	20 μM; 12.5 mg/kg body weight *in mice*
	9-*cis*-retinoic acid	0.04–10 μM; EC_50_ = 0.29 μM
	RO0721957/5	0.050 μM
	RO0264456	0.005 μM
	rosiglitazone	0.05–10 μM; EC_50_ = 1.49 μM
	RPR-5	5 μM
	rutaecarpine *and derivatives*	0.035–34.98 μM; EC_50_ = 0.27 μM
	saikosaponin A	2–8 μM
	24-(S)-saringosterol	10 μM
	SB203580	20 μM
	scutellarein	50 mg/kg body weight *in mice*
	selenium	2.5–5 μM
	serdemetan	2–5 μM
	simvastatin	10 μM
	SPF1	1 μM
	SPF2	1 μM
	soraphene A	0.03–20 μM; EC_50_ = 0.01391 μM
	24-(S)-stigmast-5-ene-3β,24-diol	10 μM
	*Cannabis sativa*-derived *stilbenoids*	2.5–3 μM
	sulfoxaflor	*u.d.*^b^ *in Aphis gossypii*
	tanshindiol C	10 μM
	taraxasterol	3–12 μM
	testosterone	0.001–0.01 μM
	tetradecylthioacetic acid	0.75% of high-fat diet *in mice*
	TO901317	0.1–25 μM; ED_50_ = 4.11 μmol/kg body weight *in mice*
	TR1	10 μM
	trichostatin A	99.2 μM; 0.5 mg/kg body weight *in mice*
	troglitazone	1 μM
	TTNPB	0.25–10 μM
	urolithin A	20 μM
	urolithin B	0.1–10 μM
	urolithin B sulfate	10 μM
	vitamin D_3_	1 μM
	vitexin	50 μM
	WAY-254011	ED_50_ = <30 μmol/kg body weight *in mice*
	Wy14643	0.05–100 μM
	bexarotene derivatives Z10 and Z36	1 μM; 40 mg/kg body weight *in mice*
	zafirlukast	2.5–5 μM
Downregulators	5CPPSS-50	20 μM
	acrolein	5–20 μM
	8-Br-cAMP	0.3 μM
	angiotensin II	0.0001–0.100 μM
	asymmetric dimethylarginine	0.5–1 μM
	atorvastatin	0.1–100 μM
	ATR-101	10–30 μM
	bisphenol A	100 μM
	*chalcone derivatives*	10 μM
	4-{[4-(4-chlorophenyl)-2-hiazolyl]amino}phenol	5 μM
	cholesterol	150 μM
	dexamethasone	0.1–2.5 μM; 8 mg/KG body weight *in rats*
	dibutyl phthalate	0.1 μM
	EGCG	100 mg/kg body weight *in mice*
	fluvastatin	0.1–100 μM
	GGPP	10 μM–200 μM
	GSK2033	0.05–5 μM
	GW6471	10 μM
	GW9662	10 μM
	desulfated holothurin A	2.68–4.47 μM
	homocysteine	50–200 μM
	*lipopolysaccharides*	1 mg/mL
	lovastatin	0.1–100 μM
	LY294002	20 μM
	methionine	17 g/kg food *in mice*
	mevalonate	100 μM
	mevastatin	0.05–50 μM
	mitotane	50 μM
	NDEA	100 mg/kg body weight *in rats*
	l,2,3,4,6-penta-*O*-galloyl-β-_d_-glucose	25–300 mg/kg body weight *in mice*
	phenylalanine-proline	1000 μM; 600 mg/kg body weight *in rats*
	pitavastatin	10 μM
	pravastatin	50 μM
	raloxifene	10 μM
	rosuvastatin	5–50 μM
	simvastatin	0.1–100 μM
	SR9243	1 μM
	tamoxifene	2.5–10 μM
	α-tocopherol	50–100 μM
	γ-tocopherol	50–100 μM
	toremifene	10 μM
	troglitazone	10 μM
	valproic acid	1000 μM
	varenicline	10 μM; 0.5 mg/kg body weight *in mice*
Stabilizers	cyclosporine A	10 μM
	diphenoquinone	0.0001–0.0005 μM
	erythrodiol	10–15 μM
	ALLN	50 μM
	leupeptin	1170 μM
	probucol	*u.c.* ^a^
	spiroquinone	0.025–0.050 μM
	testosterone	0.01 μM
	wogonin	10–40 μM
Destabilizers	brefeldin A	17.8–36 μM
	2-bromopalmitate	7.5–60 μM; IC_50_ = 15 μM
	cycloheximide	355 μM
	Gö6976	10 μM
	monensin A	10 μM
	serdemetan	2–5 μM
	tunicamycin	2.41 μM

a *u.c.* = unspecified concentration

b *u.d.* = unspecified dose

**Table 3.** Currently known modulators of ABCA transporters other than ABCA1.

Mode of modulation	Name of modulator	Effect concentration; concentration range; EC_50_; dose; ED_50_
**ABCA2**		
(Potential) substrates	cytarabine	-
	dexamethasone	-
	estramustine	-
	estradiol	-
	estrone	-
	imatinib	-
	methotrexate	-
Inducers	imatinib	*u.c.* ^b^
	methotrexate	1.28 μM
	progesterone	31.8 μM
	sulfoxaflor	*u.d.*^c^ *in Aphis gossypii*
	U18666A	5 μM
Downregulators	celecoxib	10 μM
**ABCA3**		
(Potential) substrates	cisplatin	-
	cytarabine	-
	dasatinib	-
	daunorubicin	-
	dexamethasone	-
	doxorubicin	-
	etoposide	-
	imatinib	-
	methotrexate	-
	miltefosine	-
	mitoxantrone	-
	nilotinib	-
	paclitaxel	-
	vincristine	-
Inducers	dasatinib	*u.c.* ^b^
	5-FU	50 μM
	imatinib	0.1–12.5 μM
	methotrexate	1.28 μM
	nilotinib	*u.c.* ^b^
	vitamin C	56.78 μM
Downregulators	genistein	3–9 μM
	indomethacin	2 μM
	*lipopolysaccharides*	10 μg/mL; 100 μg/mL *in chicken lungs*
	PK11195	*u.c.* ^b^
	sirolimus	2 μM
Stabilizers	C13	10 μM
	C14	10 μM
	C17	10 μM
	genistein	10 μM
	ivacaftor	1 μM
**ABCA4**		
(Potential) substrates	chloroquine	-
	hydroxychloroquine	-
	β-ionone	-
	11-*cis*-retinal	-
	13-*cis*-retinal	-
	*all-trans*-retinal	-
	*all-trans*-retinoic acid	-
	*all-trans*-retinol	-
	*N*-retinylidene-phosphatidyl-ethanolamine	-
	phosphatidyl-ethanolamine	-
Stabilizers	C3	10–20 μM
	C4	1–20 μM
	C18	10–20 μM
	lumacaftor	10–20 μM
**ABCA5**		
Inducers	atorvastatin	20 μM
	bezafibrate	10 μM
	cholesterol	100–150 μM
	GW3965	0.5 μM
	rosiglitazone	10 μM
	tacrolimus	0.04 μM
	troglitazone	10 μM
Downregulators	digoxin	2.5 g/kg body weight *in mice*
**ABCA6**		
Inducers	acitretin	1–10 mg/kg body weight *in pigs*
	lovastatin	10 μM
	mevastatin	10 μM
Downregulators	lovastatin	10 μM
	mevastatin	10 μM
**ABCA7**		
Inducers	ponasterone A	1–5 μM
	pravastatin	50 μM
	rosuvastatin	5 μM
Downregulators	cholesterol	2 mM
	digoxin	2.5 g/kg body weight *in mice*
	25-hydroxycholesterol	2.48 μM
**ABCA8**		
(Potential) substrates	p-aminohippuric acid	-
	estradiol-β-glucuronide	-
	estrone sulfate	-
	glibenclamide	-
	leukotriene C4	-
	ochratoxin A	-
	taurocholic acid	
(Potential) inhibitors	digoxin	250 μM
	dofequidar	10 μM
	glibenclamide	250 μM
	ochratoxin A	50 μM
	probenecid	1000 μM
	verapamil	1000 μM
	verlukast	100 μM
Inducers	gemcitabine	0.05–0.8 μM
	*polyethyleneglycol-block-polyactide nanoparticles*	42.04 g/kg body weight *in rats*
Downregulators	digoxin	2.5 g/kg body weight *in mice*
**ABCA9**		
Downregulators	digoxin	2.5 g/kg body weight *in mice*
**ABCA12**		
Inducers	ceramide N-hexanoyl-_d_-erythro-sphingosine	5 μM
	ciglitazone	7.5 μM
	D609 xanthate	25 μM
	_d_-DDMP	*u.c.* ^b^
	GI 251929X	10 μM
	GW610742	8 μM
	_d_-MAPP	10 μM
	_d_-NMAPPD	5 μM
	_d_-PPMP	5 μM
	_d_-PPPP	10 μM
	22-(*R*)-hydroxycholesterol	10 μM
	TO901317	10 μM
	troglitazone	7.5 μM
Stabilizers	acitretin	1–10 mg/kg body weight *in pigs*

a part from cholesterol and/or phospholipids

b *u.c.* = unspecified concentration

c *u.d.* = unspecified dose

**Table 4.** Non-exhaustive list of native ABCA transporters-expressing cell lines that have been established in the assessment of small-molecule modulators of ABCA transporters.

Cell type	Cell line name	Origin	References
**ABCA1**			
colorectal **adenocarcinoma** cells	CaCo-2	human	262,264,308,314,342,436
lung **adenocarcinoma** cells	HCC827-GR	human	337
	PC9-G2		337
renal **adenocarcinoma** cells	786-O	human	334
	A498	human	330
	ACHN	human	334,349
	HK-2	human	330
	SN12C	human	330
	OS-RC-2	human	330
**adipocytes**	3T3 L-1	mouse	255
**adrenocortical carcinoma** cells	H295R	human	333,441
	MUC-1	human	333
**astrocytes**		human	279
		mouse	229,279
		rat	281
**astrocytoma**	CCFSTTG1	human	423
peripheral **blood mononuclear** cells	PBMC	human	411
**breast cancer** cells	MCF-7	human	331
pancreatic **β-cells**	INS-1	mouse	409
**cardiomyocytes**	H9c2	rat	253
	HL-1	mouse	250
aortic **endothelial** cells	HAEC	human	263,269
endometrial **endothelial** cells		mouse	374
umbilical vein **endothelial** cells	HUVEC	human	269,364,442,496
**epithelial** cells	BEAS-B2	human	322
lung **epithelial** cells		mouse	311
pigment **epithelial** cells		human	257
mouse mammalian **epithelial** cells	MMEC	mouse	350
aortic smooth **muscle** cells	SMC	human	269
vascular smooth **muscle** cells	VSMC	*unspecified origin*	332
**fibroblasts**	*primary hip skin*	human	230,260
	WI-38 (embryonic)	human	205,246
	WI38VA13 (embryonic)	human	277
	BALB/3T3	mouse	275
	Swiss 3T3	mouse	312
**granulosa** cells		rat	443
**hair follicles**		human	282
**hepatoma**	Fu5AH	rat	318
	Hep3B	rat	231
	HepG2	human	309,342,348,379
		rat	280,312,317,367,381
	McARH7777	rat	343
**insulinoma** cells	INS-1	rat	405
**keratinocytes**		human	282
embryonic **kidney** cells		human	312
non-small cell **lung cancer** cells	A549	human	322,447
	H1650	human	400
	H1975	human	400
	H358	human	447
	PC-9/GR	human	400
**liver** cells	L02	human	406
mantle cell **lymphoma**	MCL	human	468
**macrophages**	*primary*	human	268,305,339,396,398
		mouse	306,312,313,320,329,341,360,366,439,448
	HD11	chicken	356
	J774.A1	mouse	252,254,255,259,265,271,278,289-292,384,392,393
	RAW264.7	mouse	249,312,313,321,336,339,342,352,360,365,367,369,375,376,381,385,399,402,404,406,408,410,416-419,421,424,425,438,442,448,497
	THP-1	human	231,245,249,256,268,272,275,292,308,310,312-316,321,328,335,338,339,341,342,360,363,364,366,377,384,388-397
	U937	human	307
**microglia**	*primary*	rat	355
	BV2	mouse	126,353,380
	retinal (Müller cells)	mouse	323
multiple **myeloma**	MM	human	468
**neuroblastoma**	Neuro-2a	murine	359
**neutrophils**	*primary*	human	339
**nephron** cells	A6	frog	258
**periodontal ligament stem** cells		human	325
**pheochromocytoma**	PC12	rat	280
**podocytes**		human	440
**retina** cells	ARPE-19	human	354
oral **squamous cell carcinoma** cells	CAL27	human	371
**trophoblasts**	BeWo	human	437
**ABCA2**			
**hepatoma**	HepG2	rat	179
**ovary** carcinoma	SKEM	human	238
**ABCA3**			
**cholangiocarcinoma**	M214-5FUR	human	427
**lung** epithelial cells	MLE-12	mouse	452
**hepatoma**	HepG2	rat	451
**leukemia**	*primary (acute myeloid)*	human	234
	BV173	human	234,236
	K562	human	234
	LAMA83	human	235
**lung cancer**	A549	human	241
	NCI-H1650	human	241
	NCI-H1975	human	241
**ABCA5**			
brain microvascular **endothelial** cells	HBMEC	human	428
**macrophages**	RAW264.7	mouse	321
	THP	human	321
**ABCA7**			
**fibroblasts**	BALB/3T3	mouse	205
	WI-38	human	205
**macrophages**	J774.A1	mouse	431

**Table 5.** Summary of known ATPase modulators of ABCA transporters.

Transporter	Modulator	Mode of modulation	References
ABCA1	ceramide (30 mol–%)	inhibition	201
	cholesterol (30 mol–%)	inhibition	201,499
	phosphatidylcholine (30 mol–%)	activation	201
	phosphatidylethanolamine (30 mol–%)	inhibition	201
	phosphatidylinositol (30 mol–%)	inhibition	201
	phosphatidylserine (30 mol–%)	activation	201
	sphingomyelin (30 mol–%)	activation	201
ABCA2	methyl-β-cyclodextrin (*u.c.*^a^)	activation	500
ABCA4	amiodarone (20–75 μM)	activation	138
	2-*tert*-butylanthraquinone (20–50 μM)	activation	138
	ceramide (30 mol–%)	inhibition	201
	cholesterol (30 mol–%)	inhibition	201
	dehydroabietylacetate (10–50 μM)	activation	138
	digitonin (10–180 μM)	activation	138
	*N*-ethylmaleimide (NEM; 1000 μM)	inhibition	137
	reduced glutathione (GSH; 1000 μM)	activation	137
	β-ionone (50–100 μM)	activation	138
	phosphatidylethanolamine (30 mol–%)	activation	201
	phosphatidylglycerol (30 mol–%)	activation	201
	phosphtidylinositol (30 mol–%)	inhibition	201
	11-*cis*-retinal (5–100 μM)	activation	137,138
	13-*cis*-retinal (5–100 μM)	activation	138
	ATRA (5–100 μM; EC_50_ = 10 μM)	activation	133-135,137,138
	*all-trans*-retinoic acid (20–100 μM)	activation	138
	*all-trans*-retinol (20–100 μM)	activation	133,138
	*N*-retinylidenephosphatidylethanolamine (40 μM)	activation	133
ABCA7	ceramide (30 mol–%)	inhibition	201
	cholesterol (30 mol–%)	inhibition	201
	phosphatidylcholine (30 mol–%)	activation	201
	phosphatidylethanolamine (30 mol–%)	activation	201
	phosphatidylserine (30 mol–%)	activation	201

a *u.c.* = unspecified concentration

**Table 6.** Animal models to study the functional and pathological role of ABCA transporters.

Transporter	Type	Species	Phenotype	References
**ABCA1**	knock-out	mouse	reduced cholesterol and plasma phospholipid levels decreased brain APOE levels poorly lipidated APOE	161-163 https://www.jax.org/strain/003897
	overexpression	mouse	increased lipidation of APOE	127
**ABCA2**	knock-out	mouse	reduced body weight, limb tremor, reduced sphingomyelin	https://www.jax.org/strain/033139^54^,^527^
**ABCA3**	knock-out	mouse	Knocked-out pups die within 1h after birth	186,528,529
	missense mutation	mouse	early macrophage predominant alveolitis which peaked at 8 weeks of age	530
**ABCA4**	knock-out	mouse	abnormal phospholipid composition, delayed dark adaptation	531,532
**ABCA5**	knock-out	mouse	exophthalmos and collapsed thyroid gland, early death due to cardiac insufficiency	123,131
**ABCA7**	knock-out	mouse	reduced microglia response altered phagocytosis increased β-secretase	124
	humanized	mouse	under characterization, increase Aβ load	Abca7^tm1.1(ABCA7)Pahnk^ MGI:6258226
**ABCA8**	knock-out	mouse	reduced plasma HDL	533
	adenoviral overexpression	mouse	increased plasma HDL and cholesterol	533
**ABCA12**	-	-	not described	https://www.jax.org/strain/033630
**ABCA13**	knock-out	mouse	deficits of prepulse inhibition	48
		monkey	impaired neuronal formation, neurotransmitter alterations	534

## APPENDIX

### Author information

#### Corresponding author

**Sven Marcel Stefan**, Department of Pathology, Section of Neuropathology, Translational Neurodegeneration Research and Neuropathology Lab, University of Oslo and Oslo University Hospital, Sognsvannsveien 20, 0372 Oslo, Norway;

ORCiD: 0000-0002-2048-8598

s.m.stefan@medisin.uio.no

#### Authors

**Jens Pahnke**, Department of Pathology, Section of Neuropathology, Translational Neurodegeneration Research and Neuropathology Lab, University of Oslo and Oslo University Hospital, Sognsvannsveien 20, 0372 Oslo, Norway; LIED, University of Lübeck, Ratzeburger Allee 160, 23538 Lübeck, Germany; Department of Pharmacology, Faculty of Medicine, University of Latvia, Jelgavasiela 1, 1004 Rīga, Latvia;

ORCiD: 0000-0001-7355-4213

**Pablo Bascuñana**, Department of Pathology, Section of Neuropathology, Translational Neurodegeneration Research and Neuropathology Lab, University of Oslo and Oslo University Hospital, Sognsvannsveien 20, 0372 Oslo, Norway;

ORCiD: 0000-0003-2186-8899

**Mirjam Brackhan**, Department of Pathology, Section of Neuropathology, Translational Neurodegeneration Research and Neuropathology Lab, University of Oslo and Oslo University Hospital, Sognsvannsveien 20, 0372 Oslo, Norway; LIED, University of Lübeck, Ratzenburger Allee 160, 23538 Lübeck, Germany;

ORCiD: 0000-0002-0753-6292

**Katja Stefan**, Department of Pathology, Section of Neuropathology, Translational Neurodegeneration Research and Neuropathology Lab, University of Oslo and Oslo University Hospital, Sognsvannsveien 20, 0372 Oslo, Norway;

ORCiD: 0000-0003-3544-2477

**Vigneshwaran Namasivayam**, Department of Pharmaceutical and Cellbiological Chemistry, Pharmaceutical Institute, University of Bonn, An der Immenburg 4, 53121 Bonn, Germany;

ORCiD: 0000-0003-3031-3377

**Radosveta Koldamova**, Department of Environmental and Occupational Health, School of Public Health, University of Pittsburgh, 130 De Soto Street, Pittsburgh, PA 15261, United States of America;

ORCiD: 0000-0002-6761-0984

**Jingyun Wu**, Department of Pathology, Section of Neuropathology, Translational Neurodegeneration Research and Neuropathology Lab, University of Oslo and Oslo University Hospital, Sognsvannsveien 20, 0372 Oslo, Norway;

ORCiD: 0000-0002-5137-4614

**Luisa Möhle**, Department of Pathology, Section of Neuropathology, Translational Neurodegeneration Research and Neuropathology Lab, University of Oslo and Oslo University Hospital, Sognsvannsveien 20, 0372 Oslo, Norway;

ORCiD: 0000-0002-4535-9952

## Abbreviations

5-FU: 5-fluorouracil
Aβ: amyloid-β
ABCA: ATP-binding cassette transporter subfamily A
ACAT: acyl coenzyme A cholesteryl acyl transferase
AD: Alzheimer’s disease
ADMA: asymmetric dimethylarginine
ADP: adenosine-diphosphate
ALS: amyotrophic lateral sclerosis
AMPK: cAMP-activated protein kinase
APOA1/E3/E4: apolipoprotein A1/E3/E4
APP: amyloid precursor protein
ATP: adenosine-triphosphate
BBB: blood-brain barrier
BCSFB: blood-cerebrospinal fluid barrier
BHK: baby hamster kidney
BIG1: brefeldin 1-inhibited guanine nucleotide exchange protein
BODIPY: 4,4-difluoro-4-bora-3a,4a-diaza-*s*-indacene
cAMP: cyclic adenosine monophosphate
CFTR: cystic fibrosis transmembrane conductance regulator
CHO: Chinese hamster ovary
CNS: central nervous system
CPT-cAMP: 8-(4-chlorophenylthio)-cAMP
Cryo-EM: cryogenic electron microscopy
CSF: cer-ebral spinal fluid
DIDS: 4,4’-Diisothiocyano-2,2’-stilbenedisulfonic acid
EC_50_: half-maximal effect concentration
ECD: extracellular domain
ECGC: epigallocatechin gallate
ED_50_: half-maximal effective dose
EOAD: early-onset AD
FPD5: fluorescigenic pyrazoline derivative 5
FXR: farnesoid-X-receptor
GFP: green fluorescent protein
GGPP: geranylgeraniol pyrophosphate
GSH: reduced glutathione
GWAS: genome-wide association study
HD: Huntington’s disease
HDAC2: histone deacetylase 2
HDL: high-density lipoprotein
HMG-CoA-reductase: 3-hydroxyl-3-methyl glutaryl-coenzyme A reductase
HTS: high-throughput screening
IC_50_: half-maximal inhibition concentration
LAMP1: lysosomal-associated membrane protein 1
LDLR: LDR receptor
lncRNA: long non-coding RNA
LOAD: late-onset AD
LTC_4_: leukotriene C4
LXR: liver-X-receptor
MDR: multidrug resistance
mRNA: messenger RNA
MS: multiple sclerosis
MSD: membrane-spanning domain
NBD: 7-nitro-2,1,3-benzooxadiazole *or* nucleotide binding domain
NDEA: N-nitrosodiethylamine
NEM: N-ethylmaleimide
(ox)LDL: (oxidized) low density lipoprotein
PCB29-pQ: 2,3,5-trichloro-6-phenyl-[1,4]-benzoquinone
PD: Parkinson’s disease
PDB: protein data bank
PG-J2: prostaglandin J2
PMA: phorbol 12-myristate 13-acetate
PPAR: peroxisome proliferator-activated receptor
PRDX1: peroxiredoxin 1
RAR: retinoic acid receptor
RNA: ribonucleic acid
RXR: retinoid-X-receptor
SAR: structure-activity relationships
shRNA: short-hairpin RNA
siRNA: small interfering RNA
SNP: single nucleotide polymorphism
SR-BI (Srb1): scavenger receptor B1 (also HDL receptor)
SREPB: sterol regulation element-binding protein
TKI: tyrosine kinase inhibitor
TKI: tyrosine kinase inhibitor
TM: transmembrane helix
